# Supplementary material for: Safety and immunogenicity of heterologous boost immunization with an adenovirus type-5-vectored and protein-subunit-based COVID-19 vaccine (Convidecia/ZF2001): A randomized, observer-blinded, placebo-controlled trial
Source: PLoS Med. 2022 May 26;19(5):e1003953. doi: 10.1371/journal.pmed.1003953 (PMC9187065; doi:10.1371/journal.pmed.1003953)
Supplement: S1 Study protocol — (PDF) [file pmed.1003953.s002.pdf]

Study Protocol:

Study on heterologous prime-boost immunization of recombinant COVID-19 vaccine (Ad5 vector) and RBD-based protein subunit vaccine (CHO)

Protocol Number: JSVCT115

Principle Investigator: Jing-Xin Li

Sponsor: Jiangsu Provincial Center for Disease Control and Prevention

Version: Version 1.3

Protocol Date: July 11, 2021

**Brief Title:**	Study on heterologous prime-boost immunization of recombinant COVID-19 vaccine (Ad5 vector) and RBD-based protein subunit vaccine (CHO)		
**Protocol Title:**	Safety and immunogenicity of a heterologous prime-boost immunization of recombinant COVID-19 vaccine (Ad5 vector) and RBD-based protein subunit vaccine (CHO) against COVID-19 in Chinese healthy population: a randomized, observer-blind, placebo-controlled study		
**Protocol Number:**	JSVCT115		
**Sponsor:**	Jiangsu Provincial Center for Disease Control and Prevention		
**Investigational Vaccine**	Prime: Recombinant COVID-19 vaccine (Ad5 vector)<br>Boost: RBD-based protein subunit vaccine (CHO) against COVID-19		
**Protocol Date**	July 11, 2021		
**Version:**	Version 1.3		
**Principle Investigator**	Jing-Xin Li	Chief physician	Jiangsu Provincial Center for Disease Control and Prevention

Leading Authors

Feng-Cai Zhu Chief Physician Jiangsu Provincial Center for Disease Control and Prevention

Jing-Xin Li Chief Physician Jiangsu Provincial Center for Disease Control and Prevention

Peng-Fei Jin Attending Physician Jiangsu Provincial Center for Disease Control and Prevention

Wei Chen Anhui Zhifei Longcom Biopharmaceutical Co., Ltd.

Sponsor: Jiangsu Provincial Center for Disease Control and Prevention

2021. All rights reserved. Unauthorized reproduction or use is prohibited.

Statement by Principal Investigator

I agree:

- ✧ Assume the primary investigator responsibility for this clinical study.
- ✧ Ensure that the study is carried out in accordance with the protocol and standard operating procedure (SOP) in site.
- ✧ Ensure that no changes to the protocol are made without the review and written approval of the IEC, unless necessary to eliminate immediate harm to subjects or to comply with regulatory requirements (e.g., administrative aspects).
- ✧ I am fully in control of the proper use of the investigational vaccines as described in the protocol.
- ✧ I am familiar with and will comply with the Good Practice for Quality Management of Drug Clinical Trials (GCP) and all relevant regulatory requirements.

**Brief Title:**	Study on heterologous prime-boost immunization of recombinant COVID-19 vaccine (Ad5 vector) and RBD-based protein subunit vaccine (CHO)
**Protocol Title:**	Safety and immunogenicity of a heterologous prime-boost immunization of recombinant COVID-19 vaccine (Ad5 vector) and RBD-based protein subunit vaccine (CHO) against COVID-19 in Chinese healthy population: a randomized, observer-blind, placebo-controlled study
**Investigational Vaccine**	Prime: Recombinant COVID-19 vaccine (Ad5 vector)<br>Boost: RBD-based protein subunit vaccine (CHO) against COVID-19
**Protocol Number:**	JSVCT115
**Protocol Date**	July 11, 2021
**Version:**	Version 1.3
**Principle Investigator**	Name: Jing-Xin Li<br>Professional title: Chief Physician<br>Position: Department of Vaccine Clinical Evaluation<br>Unit: Jiangsu Provincial Center for Disease Control and Prevention<br>Address: No. 172 Jiangsu Road, Nanjing, Jiangsu Province, China

	Postcode: 210009<br>Tel: 18915999772<br>fax: 025-83759529<br>E-mail: jingxin42102209@126.com
**Principle Investigator (signature)**	Date signed:

DOCUMENT HISTORY

**No.**	**Version 1.0/March 24/Original Contents**	**Version 1.1/March 30/Information of Amendment**	**Reasons for Amendment**
1	PROTOCAL ABSTRACT<br>-“Randomization and blinding” /3.5.1 Generate random coding and distribute vaccines :<br>In this study, block randomization method will be adopted, and the random codes will be generated by the random specialist with the application of SAS software. The random codes are randomly distributed in a ratio of 2:1 to generate a random coding table, in which the vaccine distribution column is covered with scratch card.	PROTOCAL ABSTRACT<br>-“Randomization and blinding” / 3.5.1 Generate random coding and distribute vaccines :<br>The subjects randomization table is generated by an independent randomization professional using SAS version 9.4 or above and imported into the Interactive Response Technology (IRT) system, accessible only to authorized personnel. Subjects, investigators and the sponsor's research management team will be blinded throughout the trial. Non-blind personnel at authorized research centers can obtain grouping information of subjects through the IRT system and use the experimental vaccine or placebo for the corresponding group based on it.	Use an Interactive Response Technology (IRT) system for randomization
2	PROTOCOL ABSTRACT-Endpoints/3.2 Endpoints-Exploratory endpoints:<br>Types of binding antibodies IgG against SARS-CoV-2 S protein at day 28 after the booster vaccination	PROTOCOL ABSTRACT-Endpoints/3.2 Endpoints-Exploratory endpoints:<br>Types of binding antibodies IgG against SARS-CoV-2 S protein at day 14, day 28 and month 6 after the booster	The detections are added at timepoints day 14 and month 6

		vaccination.	
**NO.**	**Version 1.1/March 30/Original Contents**	**Version 1.2/April 5/Information of Amendment**	**Reasons for Amendment**
1	PROTOCAL ABSTRACT/main text: the booster vaccine is an inactive COVID-19 vaccine	PROTOCAL ABSTRACT/main text: the booster vaccine is a recombinant RBD-based protein subunit vaccine (CHO)	Due to the lack of supply of inactive COVID-19 vaccine in China, the booster immunization is replaced by a recombinant protein subunit vaccine.
3	PROTOCOL ABSTRACT-Endpoints/3.2 Endpoints-Secondary endpoints: Binding antibody against S/N protein	PROTOCOL ABSTRACT-Endpoints/3.2 Endpoints-Secondary endpoints: Binding antibody against S and RBD protein	Because the recombinant COVID-19 subunit vaccine (CHO cells) contains RBD as antigen
4	PROTOCOL ABSTRACT/5.4.1 Investigational vaccine: vaccine 2: COVID-19 inactive vaccine （Verocell）<br>manufacture: Sinovac Life Science Co., Ltd.<br>Specification: 0.5ml/bottle, commercially available back, store according to instructions.	PROTOCOL ABSTRACT/5.4.1 Investigational vaccine: vaccine 2: RBD-based protein subunit vaccine (CHO cell) against COVID-19 produced by Anhui Zhifei Longcom Biopharmaceutical Co.,Ltd. It contains 25μg of NCP-RBD protein in binding region to SARS-CoV-2.	The booster immunization is replaced by a recombinant protein subunit vaccine.
**NO.**	**Version 1.2/April 5/Information of Amendment**	**Version 1.3/July 11/Information of Amendment**	**Reasons for Amendment**
	PROTOCOL	**3.1 Design procedures**	Due to the level of

	ABSTRACT-Investigational vaccine/PROTOCOL ABSTRACT-Trial design/3.1 Design procedures/3.3 Study Plan	PROTOCOL ABSTRACT-Investigational vaccine/PROTOCOL ABSTRACT-Trial design/3.1 Design procedures/3.3 Study Plan: Add "For 0-28 days regimen and 0-56 days regimen, all participants receive a boost vaccination with ZF2001 at 4 months after the 1st boost dose."	neutralizing antibody at 14 days post the 1st boost dose were moderate, we add a boost vaccination with ZF2001 at 4 months after the 1st booster dose to further boost the responses.
	PROTOCOL ABSTRACT--Endpoints/3.2 Endpoints-Timepoints: at day 14, day 28 and month 6 after booster vaccination,	PROTOCOL ABSTRACT--Endpoints/3.2 Endpoints-Timepoints: at day 14, 28 after the 1st booster vaccination, and at day 14 and month 6 after the 2nd booster vaccination.	Add a boost vaccination with ZF2001 at 4 months after the 1st booster dose.

ABBREVIATIONS

AE	Adverse Event
AR	Adverse Reaction
COVID-19	Corona Virus Disease 2019
eCRF	Electronic Case Report Form
ELISA	Enzyme-linked Immunosorbent Assay
FAS	Full Analysis Set
GCP	Good Clinical Practice
GMFI	Geometric Mean Fold Increase
GMP	Good Manufacturing Practice
GMT	Geometric Mean Titre
IEC	Independent Ethics Committee
ITT	Intent-to-treat
NIFDC	National Institute for Food and Drug Control
NMPA	National Medical Products Administration
PPS	Per Protocol Set
SAE	Serious Adverse Event
SOP	Standard Operation Procedure
SS	Safety Set

TABLE OF CONTENTS

PROTOCOL ABSTRACT.......... 3

1. Background and Principle.......... 16

1.1 Pathogen.......... 16

1.2 Disease and epidemiological background.......... 16

1.3 Basis of the study.......... 17

2. Research Purposes.......... 18

3. Trial Design.......... 19

3.1 Design procedures.......... 19

3.2 Study Endpoints.......... 19

3.2.1 Primary endpoints:.......... 19

3.2.2 Secondary endpoints:.......... 19

3.2.3 Exploratory endpoints:.......... 20

3.3 Study Plan.......... 20

3.4 Sample size calculation.......... 25

3.5 Randomization and blinding.......... 25

3.5.1 Generate random coding and distribute vaccines.......... 25

3.5.2 Maintenance of blinding.......... 26

3.5.3 Unblinding.......... 26

3.6 Criteria for pausing or early termination.......... 26

3.7 Duration of study.......... 27

4. Participants.......... 27

4.1 Participants selection.......... 27

4.2 Inclusion criteria.......... 27

4.3 Exclusion Criteria.......... 28

4.4 Withdraw from the study.......... 28

4.5 Complete of the study.......... 29

4.5.1 Complete of the safety data collection.......... 29

4.5.2 Complete of immunogenicity data collection.......... 29

4.6 Protocol violation and protocol deviation.......... 29

4.6.1 Protocol violation (including but not limited to).......... 29

4.6.2 Protocol deviation (including but not limited to).......... 30

5.0 Methods and procedures.......... 30

5.1 Participants selection.......... 30

5.2 Informed Consent.......... 30

5.3 Physical examination and screening.......... 31

5.4 Vaccine distribution and inoculation.......... 31

5.4.1 Investigational vaccine.......... 31

5.4.2 Administration.......... 31

5.4.3 Storage and transportation of investigational vaccines.......... 32

5.4.4 Combined medication/vaccine.......... 32

5.5 Safety follow up and evaluation.......... 32

5.5.1 Safety observation........ 32
5.5.2 Safety observation contents and indicators........ 33
5.5.3 Outcome of AEs........ 38
5.5.4 Relationship between AE and vaccination........ 38
5.5.5 Treatment of AEs/ARs........ 39
5.5.6 Treatment of pregnancy events........ 40
5.6 Immunogenicity evaluation........ 40
5.6.1 Samples collection........ 40
5.6.2 Preservation and transportation of samples........ 41
5.7 Data management........ 41
5.7.1 Data collection, entry and reporting........ 41
5.7.2 Verification of data records........ 41
5.7.3 Database establishment and data entry........ 42
5.7.4 Database locking........ 42
5.8 Statistics Plan and Statistical Analysis........ 43
5.8.1 Statistical plan........ 43
5.8.2 Selection of analysis data sets........ 43
5.8.3 Data statistics method........ 43
6. Monitoring of Clinical Trial........ 44
6.1 Quality assurance and quality control........ 44
6.2 Modification of clinical protocol........ 45
6.3 Scheme deviation........ 45
6.4 Confidentiality........ 45
7. Schedules........ 45
8. Ethical Approval........ 46
8.1 Ethical Review and Approval........ 46
8.2 Follow-up Review........ 46
8.3 Potential risks and minimization of risks........ 47
8.3.1 Benefits and risks........ 47
8.3.2 Vaccination........ 47
8.3.3 Blood specimen collection........ 47
9. Reference........ 错误！未定义书签。

PROTOCOL ABSTRACT

Protocol Title	Safety and immunogenicity of a heterologous prime-boost immunization of recombinant COVID-19 vaccine (Ad5 vector) and RBD-based protein subunit vaccine (CHO) against COVID-19 in Chinese healthy population: a randomized, observer-blind, placebo-controlled study
Target disease	Prevention of COVID-19 caused by SARS-CoV-2 infection
Target population	Healthy adults aged above 18 years
Sample size	120 subjects
Objectives	To evaluate the safety and immunogenicity of a heterologous prime-boost immunization of recombinant COVID-19 vaccine (Ad5 vector) and RBD-based protein subunit vaccine (CHO) against COVID-19 in Chinese healthy population.
Study site	Guanyun Center for Disease Control and Prevention
Rational and background	Vaccines are one of the most effective ways to control the COVID-19 global pandemic. Currently, there are five major platforms of COVID-19 vaccine worldwide, namely, inactivated vaccine, viral vectored vaccine, live attenuated vaccine, recombinant protein vaccine and nucleic acid vaccine. The inactivated COVID-19 vaccines are developed by China Biotechnology Technology Co., Ltd. and Sinovac Life Science Co., Ltd. and Recombinant adenovirus type 5 vectored COVID-19 vaccine (**Ad5-nCoV**) jointly developed by Military Academy of Military Medical Institute and CanSino Biologics Inc have got conditional approval on the market in China. In addition, protein-subunit-based COVID-19 vaccine (ZF2001), jointly developed by the Institute of Microbiology, Chinese Academy of Sciences and Anhui Zhifei Longcom Biopharmaceutical has been granted emergency use authorization from National Medcial Products Administration on March 2021.<br>Up to now, preliminary results from phase 3 clinical trials showed that the short-term protection rates of inactive vaccine developed by Sinovac and Ad5

	vectored vaccine developed by CanSino were 50.4% and 65.7%, respectively. Although two vaccines met the requirement of WHO for the minimum protection rate of 50%, the protection rates were moderate. Comparing to the 95% protection rate of mRNA vaccine against COVID-19 developed by Moderna and Pfizer/ Biontech, the inactive vaccine and Ad5 vectored vaccine seemed to have a lower efficacy.<br>In order to induce a robust immune response, Russia deployed a heterologous prime-boost schedule using Ad26 and Ad5 vectored COVID-19 vaccine, which has shown 91.6% efficacy against COVID-19. The United Kingdom has also announced a heterologous prime-boost immunization study of adenovirus vectored COVID-19 vaccine and mRNA vaccine, with a view to optimizing the immunization program of the existing COVID-19 vaccine and achieving better protective effect in a short time.<br>Among different vaccine modalities, heterologous strategies have shown to enhance humoral and cellular responses in several animal models. However, there is lack of regulatory guidance on heterologous (cross-platform) prime-boost immunization. In theory, the types and characteristics of the immune response induced by Ad5 vectored vaccine and protein subunit vaccine deliver antigens through different vaccine components or vector types, and the prime-boost vaccination of the two vaccines at different time points may improve the quality of the immune response, and optimize the existing immunization strategies.
Investigational vaccine	Vaccine 1 (prime): recombinant COVID-19 vaccine (adenovirus type 5 vector)<br>Manufacturer: CanSino Biologics Inc.<br>Specification: 0.5ml/bottle, 5×10^{10} viral particles/bottle, be stored and transported at 2 ~ 8℃.<br>Vaccine 2 (boost): recombinant COVID-19 protein subunit vaccine (CHO cell)<br>Manufacturer: Anhui Zhifei Longcom Biopharmaceutical Co., Ltd.<br>Specification: 25μg/0.5ml/bottle, alum-adjuvanted, be stored and transported

	at 2 ~ 8℃.<br>"Placebo": trivalent split influenza vaccine<br>Manufacturer: Dalian Aleph Biomedical Co., Ltd.<br>Specification: 0.5ml/bottle, be stored and transported at 2 ~ 8℃.<br><br>Immunization:<br>Intramuscular injection at the lateral deltoid muscle of the left upper arm.<br>0-28 days regimen: The prime dose of Ad5 vectored vaccine against COVID-19, followed by protein subunit vaccine or commercial influenza vaccine 28 days apart.<br>0-56 days regimen: The prime dose of Ad5 vectored vaccine against COVID-19, followed by protein subunit vaccine after 56 days apart.<br>For 0-28 days regimen and 0-56 days regimen, all participants receive a boost vaccination with ZF2001 at 4 months after the 1st boost dose.
Trial design	Study design:<br>This is a single center, randomized, observer-blind, placebo-controlled heterologous prime-boost immunization clinical trial.<br>Sample size:<br>60 participants in each regimen group. A total of 120 participants will be recruited.<br><br>Randomization and blinding:<br>The method of stratified block randomization is adopted in this study, and the subjects will be stratified according to age (18-59 years old and ≥60 years old) before are randomly assigned by 2:1. The randomization list is generated by an independent randomization professional using SAS version 9.4 or above and imported into the Interactive Response Technology (IRT) system. The allocation of the treatment groups is accessible only to authorized unblinded staffs. Subjects, investigators and the sponsor's research management team

	will be blinded throughout the trial. The unblinding staffs at authorized research centers can obtain grouping information of subjects through the IRT system and use the investigational vaccine or placebo for the corresponding group based on the grouping information.<br>The unblinding staffs are responsible to prepare and administrate the vaccine. The unblinding staffs do not allow to participate in other process of the trial.<br><br>Study plan:<br>120 healthy subjects aged over 18 years of age who have who have received one dose of the Ad5 vectored COVID-19 vaccine will be recruited in this study. Of them, 60 subjects will be enrolled in the "0-28 days" regimen and other 60 will be enrolled in "0-56 days" regimen. Subjects in each regimen will be randomly vaccinated with the booster dose of subunit vaccine (ZF2001) against COVID-19 or a commercial influenza vaccine in a ratio of 2:1. For 0-28 days regimen and 0-56 days regimen, all participants receive a boost vaccination with ZF2001 at 4 months after the 1st boost dose.<br>The occurrence of adverse events within 28 days post each booster vaccination, and serious adverse events within 6 months after vaccination will be observed. In addition, blood samples will be collected at baseline (at day 28 post primary dose) and at day 14, 28 after the 1st booster vaccination, and at day 14 and month 6 after the 2nd booster vaccination. For participants receiving "0-56 days" regimen, another blood sample is collected at day 0 before the 1st booster vaccination.<br><br>Study Duration:<br>Each subject will remain in this study for approximately 12 months from enrollment to discharge from the last visit.
Endpoints	Primary endpoints:<br>● Incidence of solicited adverse events within 7 days after each booster

	vaccination.<br>● GMT of neutralizing antibodies against live SARS-CoV-2 virus at day 14 after each booster vaccination.<br>Secondary endpoints:<br>Safety endpoints<br>● Incidence of adverse reactions within 28 days after each booster dose;<br>● Incidence of unsolicited AE within 28 days after each booster dose;<br>● Incidence of serious adverse events(SAE) from the 1st booster dose to the month 6 after the 2nd booster vaccination;<br>Immunogenicity endpoints:<br>● GMT of binding antibodies against SARS-CoV-2 S and RBD protein measured by ELISA at baseline (at day 28 post primary dose) and at day 14, 28 after the 1st booster vaccination, and at day 14 and month 6 after the 2nd booster vaccination.;<br>● Proportion of the participants with at least a four-fold increase of the binding antibodies against SARS-CoV-2 S and RBD protein, as compared to baseline, at day 14, 28 after the 1st booster vaccination, and at day 14 and month 6 after the 2nd booster vaccination;<br>● Geometric mean fold increase (GMFI) of binding antibodies against SARS-CoV-2 S and RBD protein measured by ELISA, as compared to baseline, at day 14, 28 after the 1st booster vaccination, and at day 14 and month 6 after the 2nd booster vaccination;<br>● GMT of neutralizing antibodies against live SARS-CoV-2 virus at 28 after the 1st booster vaccination, and at month 6 after the 2nd booster vaccination;<br>● Proportion of the participants with at least a four-fold increase of neutralizing antibodies against live SARS-CoV-2 virus, as compared to baseline, at day 14, 28 after the 1st booster vaccination, and at day 14 and month 6 after the 2nd booster vaccination;

	● Geometric mean fold increase (GMFI) of neutralizing antibodies against live SARS-CoV-2 virus, as compared to baseline, at day 14, 28 after the 1st booster vaccination, and at day 14 and month 6 after the 2nd booster vaccination;<br>Exploratory endpoints:<br>● Types of binding antibodies IgG against SARS-CoV-2 S protein at day 14, 28 after the 1st booster vaccination, and at day 14 and month 6 after the 2nd booster vaccination;<br>● Cross neutralizion of the antibodies to variants of SARS-CoV-2 at day 14 after booster vaccination;<br>● The specific memory immune cells, such as B cells and T cells, subgroups and germlines at day 14, 28 after the 1st booster vaccination, and at day 14 and month 6 after the 2nd booster vaccination.
Scheduled site visits	Visit Plan:<br>"D0-D28-M5" regimen:<br>There are a total of 9 visits, including V0 (day 28 after the prime dose, the 1st booster vaccination), V1(day 7 after 1st the booster vaccination), V2(day 14 after the 1st booster vaccination), V3(day 28 after the 1st booster vaccination), V4 (month 4 after the 1st booster vaccination, the 2nd booster vaccination), V5 (day 7 after 2nd the booster vaccination), V6 (day 14 after 2nd the booster vaccination), V7 (day 28 after 2nd the booster vaccination) and V8 (month 6 after the 2nd booster vaccination).<br>Time points of 5 blood collection:<br>At V0(day 28 after the prime dose, before the 1st booster vaccination), V3(day 28 after the 1st booster vaccination), V6 (day 14 after 2nd the booster vaccination), 20.0ml blood will be collected from each subject.<br>At. V2(day 14 after the 1st booster vaccination), V8(month 6 after the 2nd booster vaccination), 10.0ml blood will be collected from each subject. PBMC and serum will be isolated from the blood samples.

	"D0-D28-M6" regimen:<br>There are a total of 10 visits, including V-1(day 28 after the prime dose), V0 (day 56 after the prime dose, the 1st booster vaccination), V1(day 7 after 1st the booster vaccination), V2(day 14 after the 1st booster vaccination), V3(day 28 after the 1st booster vaccination), V4 (month 4 after the 1st booster vaccination, the 2nd booster vaccination), V5 (day 7 after 2nd the booster vaccination), V6 (day 14 after 2nd the booster vaccination), V7 (day 28 after 2nd the booster vaccination) and V8 (month 6 after the 2nd booster vaccination).<br>Time points of 6 blood collection:<br>At V-1 (day 28 after the prime dose), V3(day 28 after the 1st booster vaccination), V6 (day 14 after 2nd the booster vaccination), 20.0ml blood will be collected from each subject.<br>At. V0 (day 56 after the prime dose, the 1st booster vaccination), V2(day 14 after the 1st booster vaccination), V8(month 6 after the 2nd booster vaccination), 10.0ml blood will be collected from each subject. PBMC and serum will be isolated from the blood samples.
Criteria for pausing or early termination	Criteria for pausing:<br>- Occurrence of one or more ≥ grade 4 adverse reaction or serious adverse event that may be associated with vaccination;<br>- Occurrence of grade 3 adverse events with similar symptoms associated with vaccination in 10% of participants or more.<br><br>Investigators can terminate the study when any criteria for early termination is meet:<br>- One or more ≥ grade 4 adverse reaction or serious adverse event occur that may probably associated with vaccination;<br>- Occurrence of grade 3 adverse events associated with vaccination in 15% of participants or more (including injection-site reaction, systemic reaction, and

	vital signs and abnormal laboratory data);<br>- The principal investigator call for a complete termination of the trial and explain the reasons;<br>- Ethics committee call for a complete termination of the trial and explain the reasons;<br>- Administrative authority call for a complete termination of the trial and explain the reasons.
Inclusion criteria and exclusion criteria	Inclusion Criteria:<br>1. The subjects ≥ 18 years old who has completed one dose of recombinant Ad5 vectored COVID-19 vaccine;<br>2. The subjects can provide with informed consent and sign informed consent form (ICF);<br>3. The subjects are able to and willing to comply with the requirements of the clinical trial program and can complete the 6-month follow-up of the study;<br>4. Axillary temperature ≤ 37.0 ℃°.<br>5. Individuals who are in good health condition at the time of enrollment, which is determined by medical history, physical examination and clinical judgment of the investigators.<br>Exclusion Criteria:<br>1. have a medical history or family history of convulsion, epilepsy, encephalopathy and psychosis;<br>2. be allergic to any component of the research vaccines, or used to have a history of hypersensitivity or serious reactions to vaccination;<br>3. women with positive urine pregnancy test, pregnant or breast-feeding, or have a pregnancy plan within six months;<br>4. have acute febrile diseases and infectious diseases;<br>5. have severe chronic diseases or condition in progress cannot be smoothly controlled, such as asthma, diabetes, thyroid disease;

	6. Congenital or acquired angioedema / neuroedema.<br>7. have the history of urticaria 1 year before receiving the trial vaccine.<br>8. have asplenia or functional asplenia.<br>9. have thrombocytopenia or other coagulation disorders (which may cause contraindications for intramuscular injection);<br>10. have the history of immunosuppressive therapy, antiallergy therapy, cytotoxic therapy or inhaled corticosteroids (excluding corticosteroid spray therapy for allergic rhinitis, and acute corticosteroid therapy without dermatitis) over the past 6 months;<br>11. have received blood products within 4 months before injection of trial vaccines;<br>12. have received another investigational product within one month before injection of trial vaccine;<br>13. have received attenuated vaccine within 1 month before injection of trial vaccine except the recombinant Ad5 vectored COVID-19 vaccine;<br>14. have received subunit or inactivated vaccine within 14 days before the vaccination with trial vaccine;<br>15. under anti tuberculosis treatment;<br>16. not be able to follow the protocol, or not be able to understand the informed consent according to the researcher's judgment, due to various medical, psychological, social or other conditions.
Principle investigator	Name: Jing-xin Li<br>Unit: Jiangsu Provincial Center for Diseases Control and Prevention<br>Address: No. 172 Jiangsu Road, Nanjing, Chin<br>Postcode: 210009<br>Tel: 18915999772<br>Fax: 025-83759529<br>E-mail: jingxin42102209@126.com
The laboratories for	The live virus neutralizing antibody measure is by Jiangsu Provincial Center

testing	for Diseases Control and Prevention<br>Test for antibody level measurede by ELISA, pseudoviruses neutralize antibodies, B cell, T cell subgroups and germlines is by Vazyme Biotech Co.,Ltd

Table 1. “0-28 days” regimen subjects visit plan

Visit No.	V0*	V1	V2	V3	V4	V5	V6	V7	V8
Day/Month	Day 28	Day 35	Day 42	Day 56	Day 150	Day 157	Day 164	Day 178	Day 330
Visit interval	V0	V0+7 days	V0+14 days	V0+28 days	V0+4 months	V4+7 days	V4+14 days	V0+28 days	V4+6 months
Time window	±3 days	+3 days	+3 days	+3 days	+7 days	+3 days	+3 days	+3 days	±15 days
Informed consent	●				●				
Demographic information collection	●								
Physical examination and preliminary screening	●				●				
Randomization	●								
Blood collection	●(20ml)		●(10ml)	●(20ml)			●(20ml)		●(10ml)
Observation for 30 min post-vaccination	●				●				
Safety visit(AR/AE)	●	●	●	●	●	●	●	●	●
Report serious adverse event(SAE)※	●	●	●	●	●	●	●	●	●
Distribution of diary card	●				●				
Return of diary card and distribute a contact card		●				●			
Return of contact card				●				●	
Record on the Vaccination	●	●	●	●	●	●	●	●	●

and Visit Record Form									
Record the combination drug/vaccine	●	●	●	●	●	●	●	●	●

* V0 is the time for the enrollment for a booster dose

Table 2. “0-56 day” regimen subjects visit plan

Visit No.	V-1	V0*	V1	V2	V3	V4	V5	V6	V7	V8
Day/Month	Day 28	Day 56	Day 63	Day 70	Day 84	Day 180	Day 187	Day 194	Day 208	Day 360
Visit interval	V-1	V0	V0+ 7 days	V0+ 14 days	V0+ 28 days	V0+ 4 months	V4+7 days	V4+ 14 days	V0+ 28 days	V4+ 6 months
Time window	0	±3 days	+3 days	+3 days	+3 days	+7 days	+3 days	+3 days	+3 days	±15 days
Informed consent	●					●				
Demographic information collection	●									
Physical examination and preliminary screening	●	●				●				
Randomization		●								
Blood collection	●(20ml)	●(10ml)		●(10ml)	●(20ml)			●(20ml)		●(10ml)
Observation for 30 min post-vaccination		●				●				
Safety visit(AR/AE)		●	●	●	●	●	●	●	●	●
Report serious adverse event(SAE)※		●	●	●	●	●	●	●	●	●
Distribution of diary card		●				●				
Return of diary card and distribute a contact card			●				●			

Return of contact card					●				●	
Record on the Vaccination and Visit Record Form	●	●	●	●	●	●	●	●	●	●
Record the combination drug/vaccine		●	●	●	●	●	●	●	●	●

* V0 is the time for the enrollment for a booster dose.

1. Background and Principle

1.1 Pathogen

2019 Novel Coronavirus 2019(SARS-CoV-2) belongs to the genus β of coronavirus, with enveloped granules that are round or elliptic, often pleomorphic, with diameters ranging from 60 nm to 140nm. Its genetic characteristics are significantly different from those of SARS-CoV and MERS-CoV.

SARS-CoV-2 Coronaviruses belong to the genus Coronavirus in the family Coronaviridae. Coronaviruses are single-stranded RNA viruses with an envelope. They are a large group of viruses that exist widely in nature. Globally, 10% to 30% of upper respiratory tract infections are caused by HCoV-229E, HCoV-OC43, HCoV-NL63 and HCoV-HKU1, which are the second leading cause of the common cold, after rhinoviruses. Middle East Respiratory Syndrome (MERS) and Severe Acute Respiratory Syndrome (SARS), caused by coronavirus, are known to be serious infectious diseases.

The coronavirus genome encodes spike protein (S), envelope protein (E), membrane protein (M) and nucleoprotein (N) in sequence. Among them, S protein is the most important surface protein of coronavirus, which is related to the transmission ability of the virus. S protein contains two subunits: S1 and S2. S1 mainly contains receptor binding region, which is responsible for the recognition of cellular receptors. S2 contains the basic elements for the membrane fusion process. In the previous development of SARS and MERS vaccines, S protein was used as the most important candidate antigen.

1.2 Disease and epidemiological background

The COVID-19 is mainly characterized by fever, dry cough and fatigue. A small number of patients have symptoms such as nasal congestion, runny nose, sore throat, myalgia and diarrhea. Severe patients usually develop dyspnea and/or hypoxemia one week after onset, and in severe cases, rapid progression to acute respiratory distress syndrome, septic shock, refractory metabolic acidosis, haemorrhagic dysfunction and multiple organ failure, etc. It is worth noting that the course of the disease in the severe and critical patients may be moderate to low fever, or even no obvious fever. Some children and newborns showed atypical symptoms, such as diarrhea, vomiting and other digestive tract symptoms, or only mental weakness and shortness of breath.

At present, the source of infection is mainly patients infected by SARS-CoV-2. An asymptomatic infected person may also be a source of infection. The main route of transmission is by respiratory droplets and close contact is the main route of transmission. Exposure to high concentrations of aerosols in a relatively closed environment for a long period of time has the potential for aerosol transmission. SARS-CoV-2 can be isolated from feces and urine, and attention should be paid to the aerosol or contact transmission caused by feces and urine to environmental pollution. The population is generally susceptible.

1.3 Basis of the study

Currently, there are five major platforms of COVID-19 vaccine worldwide, namely, inactivated vaccine, viral vectored vaccine, live attenuated vaccine, recombinant protein vaccine and nucleic acid vaccine. The inactivated COVID-19 vaccines are developed by China Biotechnology Technology Co., Ltd. and Sinovac Life Science Co., Ltd. and Recombinant adenovirus type 5 vectored (**Ad5-nCoV**) jointly developed by Military Academy of Military Medical Institute and CanSino Biologics Inc have got conditional approval on the market in China. In addition, protein-subunit-based COVID-19 vaccine (ZF2001), jointly developed by the Institute of Microbiology, Chinese Academy of Sciences and Anhui Zhifei Longcom Biopharmaceutical has been granted emergency use authorization from National Medcial Products Administration on March 2021.

Up to now, preliminary results from phase 3 clinical trials showed that the short-term protection rates of inactive vaccine developed by Sinovac and Ad5 vectored vaccine developed by CanSino were 50.4% and 65.7%, respectively. Although two vaccines met the requirement of WHO for the minimum protection rate of 50%, the protection rates were moderate. Comparing to the 95% protection rate of mRNA vaccine against COVID-19 developed by Moderna and Pfizer/ Biontech, the inactive vaccine and Ad5 vectored vaccine seemed to have a lower efficacy.

In order to induce a robust immune response, Russia deployed a heterologous prime-boost schedule using Ad26 and Ad5 vectored COVID-19 vaccine, which has shown 91.6% efficacy against COVID-19. The United Kingdom has also announced a heterologous prime-boost immunization study of adenovirus vectored COVID-19 vaccine and mRNA vaccine, with a view to optimizing the immunization program of the existing COVID-19 vaccine and achieving better protective effect in a

short time.

Among different vaccine modalities, heterologous strategies have shown to enhance humoral and cellular responses in several animal models. However, there is lack of regulatory guidance on heterologous (cross-platform) prime-boost immunization. In theory, the types and characteristics of the immune response induced by Ad5 vectored vaccine and protein subunit vaccine deliver antigens through different vaccine components or vector types, and the prime-boost vaccination of the two vaccines at different time points may improve the quality of the immune response, and optimize the existing immunization strategies.

In this study, subjects who have completed a dose of Ad5 vectored COVID-19 vaccine will be enrolled again to receive a booster dose of COVID-19 recombinant protein subunit vaccine at 28 or 56 days after the prime dose, which forms a heterologous prime-boost immunization program. To evaluate the effect of immunogenicity after immunization, the subjects in each regimen will be randomly vaccinated with the booster dose of subunit vaccine(ZF2001) against COVID-19 or a commercial influenza vaccine in a ratio of 2:1. In addition, due to clinical trial (JSVCT093) with two doses of Ad5 vectored COVID-19 vaccine and the phase Ⅰ/Ⅱ clinical trial with three dose of recombinant protein subunit vaccine (CHO cells) in healthy adults prior to this study, their serum samples after last vaccination can be provided for parallel detection of immunogenicity data, to provide complete control data.

The clinical study protocol is formulated in accordance with the requirements of the Vaccine Administration Law, the Good Practice for Quality Management of Drug Clinical Trials (GCP), the Technical Guiding Principles for Quality Management of Vaccine Clinical Trials and the Guiding Principles for Quality Management of Vaccine Clinical Trials (Trial).

2. Research Purposes

To evaluate safety and immunogenicity of a heterologous prime-boost immunization of recombinant COVID-19 Vaccine (Ad5 Vector) and RBD-based protein subunit vaccine (CHO) against COVID-19 in Chinese healthy adults.

3. Trial Design

3.1 Design procedures

This study is a single center, randomized, observer blind, placebo-controlled heterologous prime-boost immunization clinical trial, with “0-28 days” and “0-56 days” immunization regimens. The subjects are divided into two age groups, i.e. 18-59 years and 60 years and above.

Stage 1: According to the "0-28 days" regimen, sixty eligible subjects (30 in each age group) meeting the protocol requirements will be randomly assigned in a 2:1 ratio to receive a RBD-based protein subunit vaccine (CHO) or placebo (influenza vaccine).

Stage 2: According to the "0-56 days" regimen, sixty eligible subjects (30 in each age group) meeting the protocol requirements will be randomly assigned in a 2:1 ratio to receive a RBD-based protein subunit vaccine (CHO) or placebo (influenza vaccine).

Stage 3: For 0-28 days regimen and 0-56 days regimen, all participants receive a boost vaccination with ZF2001 at 4 months after the 1st boost dose.

3.2 Study Endpoints

3.2.1 Primary endpoints:

– Incidence of solicited adverse events within 7 days after each booster vaccination.

– GMT of neutralizing antibodies against live SARS-CoV-2 virus at day 14 after each booster vaccination.

3.2.2 Secondary endpoints:

1. Safety endpoints

– Incidence of adverse reactions within 28 days after each booster dose;

– Incidence of unsolicited AE within 28 days after each booster dose;

– Incidence of serious adverse events(SAE) from the 1st booster dose to the month 6 after the 2nd booster vaccination

2. Immunogenicity endpoints

– GMT of binding antibodies against SARS-CoV-2 S and RBD protein measured by ELISA at

baseline (at day 28 post primary dose) and at day 14, 28 after the 1st booster vaccination, and at day 14 and month 6 after the 2nd booster vaccination.;

– Proportion of the participants with at least a four-fold increase of the binding antibodies against SARS-CoV-2 S and RBD protein, as compared to baseline, at day 14, 28 after the 1st booster vaccination, and at day 14 and month 6 after the 2nd booster vaccination;

– Geometric mean fold increase (GMFI) of binding antibodies against SARS-CoV-2 S and RBD protein measured by ELISA, as compared to baseline, at day 14, 28 after the 1st booster vaccination, and at day 14 and month 6 after the 2nd booster vaccination;

– GMT of neutralizing antibodies against live SARS-CoV-2 virus at 28 after the 1st booster vaccination, and at month 6 after the 2nd booster vaccination;

– Proportion of the participants with at least a four-fold increase of neutralizing antibodies against live SARS-CoV-2 virus, as compared to baseline, at day 14, 28 after the 1st booster vaccination, and at day 14 and month 6 after the 2nd booster vaccination;

– Geometric mean fold increase (GMFI) of neutralizing antibodies against live SARS-CoV-2 virus, as compared to baseline, at day 14, 28 after the 1st booster vaccination, and at day 14 and month 6 after the 2nd booster vaccination;

3.2.3 Exploratory endpoints:

– Types of binding antibodies IgG against SARS-CoV-2 S protein at day 14, 28 after the 1st booster vaccination, and at day 14 and month 6 after the 2nd booster vaccination;

– Cross neutralizion of the antibodies to variants of SARS-CoV-2 at day 14 after booster vaccination;

– The specific memory immune cells, such as B cells and T cells, subgroups and germlines at day 14, 28 after the 1st booster vaccination, and at day 14 and month 6 after the 2nd booster vaccination.

3.3 Study Plan

120 healthy subjects aged over 18 years of age who have who have received one dose of the Ad5 vectored COVID-19 vaccine in three batches consistence clinical trials will be recruited in this study. Of them, 60 subjects will be enrolled in the "0-28 days" regimen and other 60 will be enrolled in "0-56

days" regimen. Subjects in each regimen will be randomly assigned to receive the booster dose of subunit vaccine (ZF2001) against COVID-19 or a commercial influenza vaccine in a ratio of 2:1. For 0-28 days regimen and 0-56 days regimen, all participants receive a boost vaccination with ZF2001 at 4 months after the 1st boost dose.

The occurrence of adverse events within 28 days and serious adverse events within 6 months after vaccination will be observed. In addition, blood samples will be collected at baseline (at day 28 post primary dose), and at day 14, day 28 post 1st booster dose, and at day 14 and month 6 after the 2nd booster vaccination to test serum antibody levels and to profile the specific memory immune cells and antibody repertoire. Each subject will remain in this study for approximately 12 months.

In “0-28 days” regimen, participants will attend 9 visits in total, including V0 (day 28 after the prime dose, the 1st booster vaccination), V1(day 7 after 1st the booster vaccination), V2(day 14 after the 1st booster vaccination), V3(day 28 after the 1st booster vaccination), V4 (month 4 after the 1st booster vaccination, the 2nd booster vaccination), V5 (day 7 after 2nd the booster vaccination), V6 (day 14 after 2nd the booster vaccination), V7 (day 28 after 2nd the booster vaccination) and V8 (month 6 after the 2nd booster vaccination).

In “0-56 days” regimen, participants will attend 10 visits in total, including V-1(day 28 after the prime dose), V0 (day 56 after the prime dose, the 1st booster vaccination), V1(day 7 after 1st the booster vaccination), V2(day 14 after the 1st booster vaccination), V3(day 28 after the 1st booster vaccination), V4 (month 4 after the 1st booster vaccination, the 2nd booster vaccination), V5 (day 7 after 2nd the booster vaccination), V6 (day 14 after 2nd the booster vaccination), V7 (day 28 after 2nd the booster vaccination) and V8 (month 6 after the 2nd booster vaccination).

Table 1. “0-28 days” regimen subjects visit plan

Visit No.	V0*	V1	V2	V3	V4	V5	V6	V7	V8
Day/Month	Day 28	Day 35	Day 42	Day 56	Day 150	Day 157	Day 164	Day 178	Day 330
Visit interval	V0	V0+ 7 days	V0+ 14 days	V0+ 28 days	V0+ 4 months	V4+ 7 days	V4+ 14 days	V0+ 28 days	V4+ 6 months
Time window	±3 days	+3 days	+3 days	+3 days	+7 days	+3 days	+3 days	+3 days	±15 days
Informed consent	●				●				
Demographic information collection	●								
Physical examination and preliminary screening	●				●				
Randomization	●								
Blood collection	●(20ml)		●(10ml)	●(20ml)			●(20ml)		●(10ml)
Observation for 30 min post-vaccination	●				●				
Safety visit(AR/AE)	●	●	●	●	●	●	●	●	●
Report serious adverse event(SAE)※	●	●	●	●	●	●	●	●	●
Distribution of diary card	●				●				
Return of diary card and distribute a contact card		●				●			
Return of contact card				●				●	

Record on the Vaccination and Visit Record Form	●	●	●	●	●	●	●	●	●
Record the combination drug/vaccine	●	●	●	●	●	●	●	●	●

* V0 is the time for the enrollment for a booster dose.

Table 2. “0-56 days” regimen subjects visit plan

Visit No.	V-1	V0*	V1	V2	V3	V4	V5	V6	V7	V8
Day/Month	Day 28	Day 56	Day 63	Day 70	Day 84	Day 180	Day 187	Day 194	Day 208	Day 360
Visit interval	V-1	V0	V0+ 7 days	V0+ 14 days	V0+ 28 days	V0+ 4 months	V4+7 days	V4+ 14 days	V0+ 28 days	V4+ 6 months
Time window	0	±3 days	+3 days	+3 days	+3 days	+7 days	+3 days	+3 days	+3 days	±15 days
Informed consent	●					●				
Demographic information collection	●									
Physical examination and preliminary screening	●	●				●				
Randomization		●								
Blood collection	●(20ml)	●(10ml)		●(10ml)	●(20ml)			●(20ml)		●(10ml)
Observation for 30 min post-vaccination		●				●				
Safety visit(AR/AE)		●	●	●	●	●	●	●	●	●
Report serious adverse event(SAE)※		●	●	●	●	●	●	●	●	●

Distribution of diary card		●				●				
Return of diary card and distribute a contact card			●				●			
Return of contact card					●				●	
Record on the Vaccination and Visit Record Form	●	●	●	●	●	●	●	●	●	●
Record the combination drug/vaccine		●	●	●	●	●	●	●	●	●

* V0 is the time for the enrollment for a booster dose.

3.4 Sample size calculation

Sample size:

Hypothesis: GMT of vaccine group is superior to that in the control group at day 28 after the booster vaccination.

The baseline GMT level before the booster immunization is expected to be about 1:20 ($log_{10}X=1.3$) after one dose of prime vaccination with Ad5 vectored COVID-19 vaccine. After the booster dose, GMT level in the vaccine group is estimated to reach 1:60 ($log_{10}X=1.78$), while the control group remains 1:20. Assume that the Standard Deviation is about 4 ($log_{10}X=0.6$), and the allocation ratio for the vaccine group and placebo group is 2:1, then the minimal sample size to provide 80% power calculated is 40 and 20, respectively.

In each regimen group, subjects are stratified to two sub-age groups: 18-59 years and 60 years or above.

Table 3. Sample size of each immunization regimen

regimen	Sub-age group	sample size	
		Experimental vaccine group	Placebo control group
0-28 days	18-59 years	20	10
	60 years or above	20	10
0-56 days	18-59 ages	20	10
	60 years or above	20	10
Total	——	80	40

3.5 Randomization and blinding

3.5.1 Generate random coding and distribute vaccines

The study adopts the method of stratified block randomization, and subjects will be randomly assigned by 2:1 according to age group (18-59 years old and ≥60 years old). The subjects randomization table is generated by an independent randomization professional using SAS version 9.4 or above and imported

into the Interactive Response Technology (IRT) system, accessible only to authorized personnel. Subjects, investigators and the sponsor's research management team will be blinded throughout the trial. Non-blind personnel at authorized research centers can obtain grouping information of subjects through the IRT system and use the experimental vaccine or placebo for the corresponding group based on it.

3.5.2 Maintenance of blinding

Subjects, safety observers, laboratory testers will be blinded.

Those who administer, prepare and administer vaccines are unblinded staff and must sign a blinding maintenance agreements to ensure that any documents of the unblinding information are only accessible for the authorized non-blinded staff. The labels on vaccine syringe will be covered with a study number label after the preparation of the vaccine and put it ready to use.

3.5.3 Unblinding

The investigators must not disrupt the blind study of the vaccine/placebo unless the study information is medically necessary for the subjects. In the event of a medical emergency, the principal investigator should be contacted as far as possible before disrupting the study vaccine/placebo blinding to discuss the need for an urgent unblinding.

Blinding will be uncover when completing the initial analysis of safety and immunogenicity 28 days after the booster dose, but the subjects and safety observers will remain blinded.

3.6 Criteria for pausing or early termination

Criteria for pausing:

- Occurrence of one or more ≥ grade 4 adverse reaction or serious adverse event that may be associated with vaccination;
- Occurrence of grade 3 adverse events associated with vaccination in 10% of participants or more.

- Administrative authority call for a complete termination of the trial and explain the reasons.

investigators can terminate the study when any criteria for early termination is meet:

- One or more ≥ grade 4 adverse reaction or serious adverse event occur that may probably associated with vaccination;
- Occurrence of grade 3 adverse events associated with vaccination in 15% of participants or more (including injection-site reaction, systemic reaction, and vital signs and abnormal laboratory data;
- The principal investigator call for a complete termination of the trial and explain the reasons;
- Ethics committee call for a complete termination of the trial and explain the reasons;

3.7 Duration of study

It will take about 12 months for each participant from recruiting to completing the last visit.

4. Participants

4.1 Participants selection

Healthy people aged 18 and above who have been vaccinated of a dose recombinant COVID-19 Vaccine (Ad5 Vector), are selected as the target population, and informed in writing by informed consent approved by the ethics committee. On the premise that the volunteers themselves will sign the informed consent, they can only participate in the study after passing the physical examination and the following inclusion and exclusion criteria.

4.2 Inclusion criteria

- The subjects over 18 years old who has completed one dose of recombinant Ad5 vectored COVID-19 vaccine;
- The subjects can provide with informed consent and sign informed consent form (ICF);
- The subjects are able to and willing to comply with the requirements of the clinical trial program and can complete the 6-month follow-up of the study;
- Axillary temperature ≤ 37.0 ℃.
- Individuals who are in good health condition at the time of entry into the trial as determined by medical history, physical examination and clinical judgment of the investigators and meet the requirements of these products immunization.

4.3 Exclusion Criteria

– Have the medical history or family history of convulsion, epilepsy, encephalopathy and psychosis;

– Be allergic to any component of the research vaccines, or used to have a history of hypersensitivity or serious reactions to vaccination;

– Women with positive urine pregnancy test, pregnant or breast-feeding, or have a pregnancy plan within six months;

– Have acute febrile diseases and infectious diseases;

– Have severe chronic diseases or condition in progress cannot be smoothly controlled, such as asthma, diabetes, thyroid disease;

– Congenital or acquired angioedema / neuroedema.

– Have the history of urticaria 1 year before receiving the trial vaccine.

– Have asplenia or functional asplenia.

– Have thrombocytopenia or other coagulation disorders (which may cause contraindications for intramuscular injection);

– Have the history of immunosuppressive therapy, antiallergy therapy, cytotoxic therapy or inhaled corticosteroids (excluding corticosteroid spray therapy for allergic rhinitis, and acute corticosteroid therapy without dermatitis) over the past 6 months;

– Have received blood products within 4 months before injection of trial vaccines;

– Have received another investigational product within 1 month before injection of trial vaccine;

– Have received attenuated vaccine within 1 month before injection of trial vaccine except the recombinant Ad5 vectored COVID-19 vaccine;

– Have received subunit or inactivated vaccine within 14 days before the vaccination with trial vaccine;

– Under anti tuberculosis treatment;

– Not be able to follow the protocol, or not be able to understand the informed consent according to the researcher's judgment, due to various medical, psychological, social or other conditions.

4.4 Withdraw from the study

Participants have the right to withdraw from the study at any time during the study period, and the investigators should record the reason of withdraw:

– Loss of contact, early withdraw of the study;

– Request to withdraw without any reason;

– Withdraw for reasons unrelated to the study, such as long-term departure, relocation, etc., and the specific reason for withdrawal should be recorded;

– Withdrawal for reasons related to the study, such as intolerance of adverse reactions, intolerance of biological specimen collection, etc., and the specific reason for withdrawal should be recorded. If a participant withdraw because of AE or SAE, investigators should follow up the participant until the resolve of AE or SAE.

– Participants can require a complete withdraw from the study, all study behaviors can be stopped, including vaccination, biological specimen collection and safety observation. The data before withdrawal will not be used for analysis if he or she require so. If the participants allow the investigators use the data collected before the withdrawal, the data can be included in analysis;

– Participants can require a partially withdraw from the study, such as refuse to vaccination or blood drawn only, but still participate in other procedures during the follow-up.

4.5 Complete of the study

4.5.1 Complete of the safety data collection

The participants who receive experimental vaccine, and complete safety observation within 28 days post each booster dose, and reported SAEs through the study will be considered as complete of the safety data collection.

4.5.2 Complete of immunogenicity data collection

The participants who meet the inclusion and do not meet any exclusion criteria, take the vaccination, and complete the visits and blood collection required by the study protocol will be considered as complete of the immunogenicity data collection.

4.6 Protocol violation and protocol deviation

4.6.1 Protocol violation (including but not limited to)

– No informed consent singed by the participant;

– The enrolled participant does not meet the all the inclusion criteria or meet one or more exclusion criteria;

– The participant received incorrect intervention;

– The participant received a vaccine fail to meet the requirements;

– Any other reasons identified by the investigators and confirmed by the principal investigator.

4.6.2 Protocol deviation (including but not limited to)

- Beyond the visiting time window;
- Low compliance of participants, and the participants do not complete the blood sample collection;
- Serious adverse events do not report in time (SAE);
- Participants are treated with unallowed drugs (intramuscular, oral or intravenous corticosteroids for ≥ 2mg/kg/days, continuous use for ≥ 14 days, or other immunosuppressants);
- The interval between vaccination with other vaccines is insufficient;
- Other reasons considered as protocol deviation by the principal investigator.

Investigators or monitors should report any protocol violation or deviation to principal investigator or coordinators as soon as possible after knowing it by fax or e-mail. Protocol violation should also be reported to the ethics committees.

5.0 Methods and procedures

5.1 Participants selection

Healthy people aged 18 and above who have been vaccinated of a dose recombinant COVID-19 Vaccine (Ad5 Vector), are selected as the target population.

5.2 Informed Consent

When obtaining and recording informed consent, researchers should abide by relevant regulations, GCP and the ethical principles stipulated in the Declaration of Helsinki. Before the start of the study, the investigators should obtain written approval/consent from the ethics review committee for the informed consent form and other documents provided to the subjects.

Before participating in this clinical study, researchers should explain the contents of the informed consent form to the subjects and/or their witnesses, and the subjects and/or their witnesses should be given sufficient time to consult the details of the study before signing the informed consent form. When explaining the information of informed consent to multiple persons, each subject and/or witness should be given the opportunity to ask the investigators individually before signing the informed consent form.

Researchers should keep the informed consent form signed by each subject, and provide the subject with a copy of the signed name and date of the informed consent form.

5.3 Physical examination and screening

The subjects' body temperature will be measured before enrollment, and HCG detection will be performed on pre-menopausal women.

According to the "inclusion and exclusion criteria", the interviewers conduct medical history inquiry and screening. Only those who passed the screening can be enrolled and participate in the randomization.

5.4 Vaccine distribution and inoculation

The unblind staff, who are responsible for vaccine preparation will assign the allocated treatment to the subjects according to the random number generated by an independent statistical party. After the preparation of the vaccine, they hand the ready-to-use syringes to the vaccination nurse, who will administrate the vaccination.

First aid drugs such as epinephrine hydrochloride and first aid equipment such as simple ventilator and ECG monitor should be provided at the vaccination site.

5.4.1 Investigational vaccine

Investigational vaccine 1 (prime dose): the recombinant New Coronavirus vaccine (adenovirus vector) produced by CanSino Biologics Inc, liquid dosage form, 0.5 ml/ bottle, contains recombinant replication defective human 5 adenovirus 5×10^{10} virus particles expressing New Coronavirus S protein.

Investigational vaccine 2 (booster dose): the New Coronavirus recombinant subunit vaccine (CHO cell) produced by Anhui ZhiFei Longcom Biopharmaceutical Co., Ltd., "Zhi Ke Wei De", 0.5ml/ bottle, contains 25 μg NCP-RBD protein of New Coronavirus spike protein binding region NCP-RBD .

"Placebo": a Trivalent split influenza vaccine produced by Dalian Aleph Biomedical Co., Ltd., liquid dosage form, 0.5 ml/ bottle, contains 15 μg of H1N1, 15 μg of H3N2 and 15 μg of B-line hemagglutinin.

5.4.2 Administration

Subjects are vaccinated according to the immunization procedure. The vaccine should be fully shaken before use, and should be used immediately after opening. In case of cracks, unclear or invalid labels, or abnormal appearance of the vaccine, it should not be used.

The vaccines inject intramuscularly at the attachment of the lateral deltoid muscle of the upper arm, with priority given to the left arm.

5.4.3 Storage and transportation of investigational vaccines

(1) Vaccine storage: investigational vaccines must be stored in a safe and locked place, and must not be contacted by unauthorized persons. The temperature of vaccine storage place should be controlled in the range of 2-8℃ to prevent freezing; the storage temperature of vaccine should be recorded once in the morning and afternoon of each working day.

(2) Vaccine transportation: vaccines are transported from the research site to the vaccination site, from the vaccination site to the research site, and stored in the refrigerator or freezer. Each cold chain equipment is equipped with a thermometer. The vaccine administrator records the temperature every 30 minutes, and fills in the transportation and storage temperature records in detail. The storage temperature (2-8℃) must be kept during transportation. Any over temperature must be reported to the site responsible researcher or project coordinator for instructions. All vaccine transportation processes must be recorded.

5.4.4 Combined medication/vaccine

When the medical events happen during the study period, the participant are allowed to carry out the appropriate medical treatment, but the medical treatment should be recorded in time.

Other vaccination is not recommended except for emergency during the research period, such as rabies vaccine, tetanus vaccine, or other emergent vaccination need. Any vaccine used is required to be recorded during the study period.

5.5 Safety follow up and evaluation

5.5.1 Safety observation

After vaccination, the participants will stay at the clinic for 30-minute safety observation. The trained researchers should systematically observe each subject, record the local and systemic reactions within 30 minutes, and record the severity.

The participants are followed for the next a few days, and asked to record the safety observation by

themselves on the diary card till 7 days after the vaccination. From the day 8 to the day 28 after vaccination, the adverse events are recorded passively. At the clinic visit, the researcher will retrospectively check and verified the adverse events recorded during the safety observation. From day 28 to month 6 after vaccination, the subjects are asked to report only serious adverse events during this period.

5.5.2 Safety observation contents and indicators

. Adverse events from the clinical trial are graded according to the guiding principles for the classification of adverse events in clinical trials of preventive vaccines (NMPA [2019] No. 102), as follows: (table 4-5)

Table 4 Grading of (local) AEs at injection site

Symptoms	Grade 1	Grade 2	Grade 3	Grade 4
**Pain**	Do not affect or slightly affect physical activity	affect physical activity	Affect daily life	Loss of basic self-care ability or hospitalization
**Induration*, swelling** (optional)** #	Diameter 2.5~＜5 cm or area 6.25~＜ 25 cm2 and does not affect or slightly affect daily life	Diameter 5~＜10 cm or area 25~＜100 cm2 or affect daily life	Diameter ≥ 10 cm or area ≥ 100 cm2 or ulceration or secondary infection or phlebitis or aseptic abscess or wound drainage or seriously affect daily life	Abscess, exfoliative dermatitis, dermal or deep tissue necrosis
**Rash*,Redne ss** (optional)** #	Diameter 2.5~＜5 cm or area 6.25~25 cm2 and does not affect or slightly affect daily life	Diameter 5~＜10 cm or area 25~＜100 cm2 or affect daily life	Diameter ≥ 10 cm or area ≥ 100 cm2 or ulceration or secondary infection or phlebitis or aseptic abscess or wound drainage or seriously	Abscess, exfoliative dermatitis, dermal or deep tissue necrosis

			affect daily life	
**Itch**	Itching at the vaccination site, relieved by itself or within 48 hours after treatment	Itching at the vaccination site, which does not resolve within 48 hours after treatment	Affect daily life	NA
**Cellulitis**	NA	Non-injectable treatment is required (e.g. oral antibacterial, antifungal, antiviral therapy)	Intravenous treatment is required (e.g. intravenous antibacterial, antifungal, antiviral therapy)	Sepsis, or tissue necrosis, etc.

Note: *: in addition to directly measuring the diameter for grading and evaluation, the progress of the measurement results should also be recorded.

** the maximum measuring diameter or area should be used.

the evaluation and grading of induration and swelling, rash and redness should be based on the functional level and the actual measurement results, and the indicators with higher classification should be selected.

Table 5 Grading for systemic adverse events.

Sign	Grade 1	Grade 2	Grade 3	Grade 4
**Fever [Axillary temperature（℃）] ＞14 years old**	37.3～＜38.0	38.0～＜38.5	38.5～＜39.5	≥ 39.5, last more than 3 days
**Gastrointestinal system**				
Diarrhea	Mild or transient, 3 to 4 times a day,	Moderate or persistent, 5-7 times a day,	＞7 times/day, abnormal stool, or	Hypotension shock, hospitalization

	abnormal stool, or mild diarrhea last less than 1 week	abnormal stool characteristics, or diarrhea ＞1 week	hemorrhagic diarrhea, orthostatic hypotension, electrolyte imbalance, need intravenous infusion ＞2L	required
Dysphagia	Mild discomfort when swallowing	Diet is restricted	Diet and conversation are very limited; you can't eat solid food.	Can't eat liquid food; need parenteral nutrition.
Anorexia	Loss of appetite, but no reduction in food intake	Loss of appetite, reduced food intake, but no significant weight loss.	Loss of appetite and weight loss	Need for intervention (e.g. gastric tube feeding, parenteral nutrition)
Vomiting	1- 2 times/24 hours and does not affect the activity	3- 5 times/24 hours or activity is restricted	＞6 times/24 hours or need intravenous rehydration	Hypotension shock requires hospitalization or other means of nutrition
Nausea	Transient (<24 hours) or intermittent and food intake is normal	Continued nausea leads to reduced food intake (24-48 hours)	Persistent nausea results in almost no food intake (> 48 hours) or requires intravenous fluid replacement	Life-threatening (eg hypotension shock)
**Musculoskeletal and connective tissue**				
Non-injection-site muscle pain	Does not affect daily activities	Slightly affect daily activities	Severe muscle pain that seriously affects daily activities	Emergency or hospitalization
Arthritis	Mild pain with	Moderate pain with	Severe pain with	Permanent and/or

	inflammation, erythema, or swelling of joints; but does not interfere with function	inflammation, erythema, or swelling of joints; impairs function but does not affect daily activities	inflammation, erythema, or joint swelling; affecting daily activities	disabling joint injury
Arthralgia	Mild pain without hindering function	Moderate pain; need analgesics and/or pain that impedes function but does not affect daily activities	Severe pain; need analgesics and/or pain affecting daily activities	Disability pain
**nervous system**				
Headache	Does not affect daily activities and requires no treatment	Transient, slightly affects daily activities and may require treatment or intervention	Seriously affects daily activities and requires treatment or intervention	Intractable and requires emergency or hospitalization
Syncope	Close to syncope without losing consciousness (pre-syncope)	Loss of consciousness without treatment	Loss of consciousness and needs treatment or hospitalization	NA
**The spirit system**				
Insomnia	Mild difficulty in falling asleep, not affecting or slightly affecting daily life	Moderate difficulty in falling asleep, affecting daily life	Serious difficulty in falling asleep, seriously affecting daily life, requiring treatment or hospitalization	NA
**Skin and subcutaneous tissue**				
Non-injection-site	Slightly itchy without	Itching affects daily	Itching makes it	NA

itching (no skin lesions)	affecting or slightly affecting daily life	life	impossible to carry on daily life.	
Abnormal skin and mucosa	Erythema/itching/color change	Diffuse rash/macular papule/dryness/desquamation	Blister/exudation/desquamation/ulcer	Exfoliative dermatitis involving mucous membrane, or erythema multiforme, or suspected Stevens-Johnsons syndrome
**The respiratory system**				
Cough	Transient, without treatment	Persistent cough, effective treatment	Paroxysmal cough, uncontrollable treatment	Emergency or hospitalization
**The immune system**				
Acute allergic reaction **	Local urticaria (blister) without treatment	Local urticaria requiring treatment or mild angioedema without treatment	Extensive urticaria or angioedema requiring treatment or mild bronchospasm	Anaphylactic shock or life-threatening bronchospasm or throat edema
**Others**				
Fatigue	Does not affect daily activities	Affects normal daily activities	Seriously affects daily activities and cannot work	Emergency or hospitalization
Non-injection-site pain# (Specify the location when reporting)	Minor pain that does not affect or slightly affect daily life	Pain affects daily life	Pain can't carry on daily life	Disability pain, loss of basic self-care ability

Note: * refers to type I hypersensitivity.

# Refers to Non-injection-site pain other than muscle pain, Arthralgia and headache

General principles for the grading for other adverse events

The intensity of adverse events not mentioned in the rating table shall be evaluated according to the following criteria:

Grade 1	Grade 2	Grade 3	Grade 4	Grade 5
Mild: Short-term (< 48 hours) or mild discomfort, no influence on activities, treatment not indicated	Moderate: Mild or moderate restricted activities, presentation indicated possibly, treatment not indicated or mild treatment indicated	Severe: Significant restricted activities, presentation and treatment indicated, hospitalization indicated possibly	Critical: Life-threatening possibly, severely restricted activities, intensive care indicated	Death

5.5.3 Outcome of AEs

The outcomes of ARs/AEs include: (1) Recovery; (2) Not yet recovered; (3) Recovered but sequelae; (4) Death; (5) Loss of visit.

5.5.4 Relationship between AE and vaccination

Investigators should make the best interpretation of AE, and assess the possible causal relationship between vaccination and reactions (such as history of underlying diseases, combined treatment of causation). This applies to all AEs, including severe ones and non-severe ones. The assessment of causality will be reasonably explained in the following or more aspects of the event: The similar reaction to the solution was observed in the past; identical events of similar types solution have been reported in the literature; the incident occurred along with the time of the vaccination, and again after the secondary vaccination According to definitions, all the solicited AE (that is, the local adverse event of the collection of the report) will be considered to be related to vaccination. The causal relationship of AE should be evaluated according to the following questions, and according to your judgment, the reasonable possibility of relationship between AE and vaccination is caused by the vaccination:

1. Related: there is a suspicion that a link between vaccine and the AE (do not need to be determined);

the vaccine has a reasonable potential for promoting the AE.

2. Unrelated: there is no suspicion that a link exists between vaccine and the AE; there are other more likely causes, and vaccination has not been suspected to promote the AE.

5.5.5 Treatment of AEs/ARs

An adverse event (AE) is any untoward medical occurrence in a patient or clinical trial participant administered with a pharmaceutical product and which does not necessarily have a causal relationship with this treatment.

Adverse reactions (AR): unexpected or harmful reactions in the process of vaccination according to the prescribed dose and procedure, usually related to vaccination.

Serious adverse event (SAE): refers to the following important medical events, whether or not related to the vaccine clinical trial, including: 1) death; 2) life threatening; 3) hospitalization or prolonged hospitalization; 4) permanent or significant disability / loss of function; 5) congenital abnormality or birth defect; 6) severe adverse event It may lead to other important medical events, such as those listed above without treatment.

Suspected Unexpected Serious Adverse Reaction (SUSAR): Suspected adverse reactions refer to the adverse reactions of subjects at any dose that have nothing to do with the purpose of the medication. After analysis, it is considered that the relationship with the drug is at least likely to be related; Unexpected refers to adverse reactions. The nature, extent, consequences, or frequency are different from the expected risks described in the previous plan or other related materials (such as the investigator's manual and instructions).

If subjects have any clinically significant disease/event after vaccination, it should be reported to the investigators as soon as possible. The investigators should follow up the adverse reaction/event until the symptoms disappear or the symptoms stabilize. When the investigators deem it necessary, treatment will be provided unconditionally to relieve the pain caused by the adverse reaction/event for the subjects. All medical treatments will be recorded at each follow-up.

In the event of a serious adverse event/reaction, the investigators should take necessary measures quickly, fill in the "Serious Adverse Event Report Form" within 24 hours, and report it to the principal investigator in the form of fax or E-mail.

5.5.6 Treatment of pregnancy events

Vaccination of the trial is not allowed during pregnancy. Before vaccination, the subjects will be given urine pregnancy test, and those who are positive in the urine pregnancy test should not be included in the group. If a pregnancy event occurs within 6 months of the visit, the Pregnancy Case Survey Form needs to be filled out.

5.6 Immunogenicity evaluation

5.6.1 Samples collection

"0-28 days" immunization regimen:

At V0 (day 28 after the prime dose and before the booster dose) and V3 (day 28 after the booster dose), 20 ml of venous blood will be collected by vacuum anticoagulant blood collection vessel. PBMC and serum will be separated to detect the antibody level, immune cell differentiation and antibody spectrum induced by the vaccine.

In V2 (day 14 after the booster dose) and V4 (month 6 after the booster dose), 10ml of venous blood will be collected by vacuum anticoagulant blood collection vessel, and the serum will be separated to detect the antibody level, immune cell differentiation and antibody spectrum induced by the vaccine.

"0-56 days" immunization regimen:

At V-1 (day 28 after the prime dose) and V3 (day 28 after the booster dose), 20 ml of venous blood will be collected by vacuum anticoagulant blood collection vessel. PBMC and serum will be separated to detect the antibody level, immune cell differentiation and antibody spectrum induced by the vaccine.

At V0 (day 56 after the prime dose and the booster dose), V2 (day 14 after the booster dose) and V4 (month 6 after the booster dose), 10ml of venous blood will be collected by vacuum anticoagulant blood collection vessel, and the serum will be separated to detect the antibody level, immune cell differentiation and antibody spectrum induced by the vaccine.

The serum samples of this clinical trial will be used to evaluate the immune response level of the research vaccine. For other studies, the approval of the ethics committee and the consent of the subjects are required.

5.6.2 Preservation and transportation of samples

Unified operation standards are adopted in the process of preservation and transportation. The storage temperature of serum should be - 20℃ and below, and it should be transported to the testing laboratory in time. The separation, transportation and preservation of BPMC used for the differentiation of immune cell population and the detection of antibody spectrum are operated by the third party laboratory according to the standard operating procedures.

5.7 Data management

5.7.1 Data collection, entry and reporting

Vaccination and visit records and other original data should be clearly recorded, which should be filled in with a black signature pen. The errors of the original records should not be wiped or covered, but should be crossed off, put the corrected data aside, and signed and dated by the investigators.

Fill in all the case report forms according to the protocol. Case report form is used to record the data of clinical trials. It is an important part of clinical trials and research reports. It should be filled in clearly and completely. It is required to fill in with a black signature pen. The mistakes should not be erased or covered by the original record. Instead, a horizontal line should be drawn on it, and the corrected data should be indicated in the blank beside it. The revised researcher should sign his name and indicate the date.

According to the requirements of the scheme, data collection, biological sample collection and examination should be carried out in each visit time window, and the original documents and records should be complete, and the examination conclusions should be entered into the case report form (CRF) in time.

5.7.2 Verification of data records

The quality controller should check the data record regularly and irregularly until the CRF is completed. Before withdrawing the CRF, the quality controller should carefully check the CRF number of the subjects, the number of pages of each CRF and the necessary signature of the researcher. The main content of quality control should focus on the following links: signing of informed consent, volunteer

screening, immunization, management of experimental vaccine, safety observation, collection and preservation of immunogenic samples, etc. the consistency of research data and original data should be focused on, and manual verification should be carried out. The verification results shall be recorded. The transfer of CRF and other research materials should be documented.

5.7.3 Database establishment and data entry

Establishment of database:

The personnel in charge of data management shall establish the database structure and check procedures according to CRF, ensure that the database can be correctly converted to SAS file format, and modify and confirm the database structure through trial input.

Further verification of CRF:

Before entering CRF, data management personnel should check CRF again, mainly to see if there are omissions and obvious errors.

Data entry:

After the training of data entry personnel, data entry is carried out by two persons and two computers.

Data comparison and examination:

Check the consistency of the database data independently completed by the two people, report the inconsistent values and information, and then check the original data item by item to correct until the two databases are consistent. Use the computer program that has been written and confirmed to logically check the data, issue the query form and ask the researcher to confirm and then modify the database until there is no doubt. It is necessary to select a certain number of CRF randomly according to a certain proportion to control the quality of the database, and compare with the data in the database manually, so as to ensure that the data in the database is consistent with the CRF content.

5.7.4 Database locking

Before statistical analysis, it is necessary to conduct a check and clean of the database. The analyzed population will be set according to the definition of the data sets, including the FAS set, PPS set and safety analysis data set, and to determine the deviation from the regimen and its impact on the analysis data set. After the database is cleaned, the database will be locked, and the statistical analysis plan is locked at the same time.

5.8 Statistics Plan and Statistical Analysis

5.8.1 Statistical plan

The statistical analysis of this study will be completed in two stages. After the subjects complete the visit 28 days after the booster dose immunization, the study database will be recorded, reviewed and locked for the first stage statistical analysis.

The safety and immunogenicity data after 28 days till 6 months after immunization will be collected, reviewed and locked for the second stage statistical analysis and summary.

5.8.2 Selection of analysis data sets

Safety data set (SS):

The safety evaluation should be conducted for all participants who receive vaccines after randomization. Data violating the protocol should not be eliminated.

Immunogenicity data set:

Full Analysis Set (FAS): It is defined as ideal participant population determined according to the ITT (Intention-to-treat analysis) principle, all participants who meet the inclusion / exclusion criteria, and are randomized and given vaccine and have at least one post-immunization blood test results are included in the FAS.

Per-Protocol Set (PPS): It is a subset of FAS. Participants in this set are more compliant with the protocol, experience no major protocol violation, comply with all inclusion criteria / exclusion criteria, and complete the vaccination within the time window as required in the protocol and all blood samplings are included in the PPS set. Participants who violate the trial protocol, such as poor compliance or lost to follow-up, and those who suffer intercurrent SARS-CoV-2 infection will not be included in this analysis set.

In this trial, the FAS will be used as the primary analysis set. However, PPS should be analyzed simultaneously. Any inconsistency between PPS and FAS analysis results should be discussed in the report.

5.8.3 Data statistics method

During statistical analysis, first, the number of completed cases and drop-out cases should be checked. Then demographic and baseline characteristics of each group at enrollment should be analyzed to investigate intergroup comparability. Efficacy evaluation of vaccine includes the determination of evaluation indicators and intergroup comparison of efficacy. Safety evaluation includes the statistics of clinical ARs/AEs.

Participant elimination criteria: participants don’t meet the inclusion criteria; data and information after vaccination are not followed up; information and data after randomization are seriously missing; participants meet exclusion criteria but are not withdrawn; participants receive wrong vaccination or

incorrect dose.

Safety analysis in this trial mainly includes descriptive analysis of the incidence of ARs/AEs. χ^2 test may be carried out for intergroup comparison, and Fisher's exact test may be performed if necessary. After immunization, the number of case-times and person-times of local AEs in the high-dose group will be calculated (with conventional calculation method). The number of person-times will be calculated based on the highest severity in both arms, and the number of case-times will be calculated based on the cumulative local AEs actually occurring at the vaccination site. Logarithmic transformation is required for analysis of immunogenicity indicator of antibody level which should be expressed as GMT, standard deviation, median, maximum and minimum and 95% confidence interval. Classification indicators will be compared between groups. Antibody seroconversion rate will be analyzed by χ^2 test and Fisher's exact test may be used if necessary. Study data at different time points will be analyzed with statistical analysis for repeated measurement data.

SAS 9.4 is adopted for all statistical analyses with two-sided test. The P value is directly calculated while carrying out Fisher's exact test when test statistics and corresponding P values are given, and in case of $P \leq 0.05$, the difference is statistically significant.

6. Monitoring of Clinical Trial

6.1 Quality assurance and quality control

Carry out on-site quality control in strict accordance with the relevant requirements of Good Clinical Practice (GCP).

Investigators in some positions are qualified as physicians or above. Prior to the clinical trial, they will be trained in the clinical protocol and all trial procedures, including information about the trial vaccine, procedures for obtaining informed consent, operating procedures for each position, and procedures for reporting adverse reactions/events.

The data of each subject is reviewed at each stage of the clinical trial to ensure that the content of the clinical trial meets the requirements of the protocol and that the obtained data are complete and reliable. The quality controller controls the whole process of the clinical trial.

All the work on site are carried out strictly in accordance with the clinical trial field operation manual. Each subject records the "*Diary Card*" by themselves, follows up and retrospectively investigats by the researcher, and reviews and guides the filling in of the "*Diary Card*".

The quality controller shall conduct a comprehensive check on the original data, and after training, a special person shall enter the data of eCRF. The double entry method shall be adopted and completed by two people independently.

Calibration or standardization of the instruments used in this clinical trial.

6.2 Modification of clinical protocol

After this plan is approved by the Ethics Committee, if there is any major modification in the implementation process, it shall be reported to the Ethics Committee for approval before it can be implemented. The investigators shall not execute any deviation or change without the consent of the Sponsor and prior review and written approval of the Ethics Committee (EC).

Any changes to the scheme, whether material or non-material, are required to be in writing. EC approval is required to identify substantive protocol changes that would affect the safety of subjects, the scope of the study, or the scientific quality of the study.

6.3 Scheme deviation

The investigators shall carry out the clinical trial according to protocol approved by the ethics committee and the provisions of GCP. During the trial, the researcher shall not deviate from the protocol unless the harm to the subjects is eliminated.

The research center shall record all protocol deviations in the original data of subjects, including but not limited to the occurrence time of protocol deviation, discovery time, event description and measures, etc. In case of serious protocol deviations, the main researchers should be informed in time and report to the IEC.

6.4 Confidentiality

The sponsor, investigators, IEC, or a fully authorized representative of regulatory authority should have the right to obtain data related to the clinical trial, but relevant content cannot be used for any other clinical trials, nor can it be disclosed to any other individuals or entities.

Investigators must sign a confidentiality agreement to confirm that he/she knows and agrees to hold the information of this study confidential.

Investigators and other study personnel should keep all information provided by the sponsor and all data/information generated at the study site (except for medical records of participants) confidential. Such information and data should not be used for any purposes other than the study. This restriction does not apply to: (1) study information is not disclosed because of violations by investigators and researchers; (2) study information is disclosed only to the IRB/IEC for the purpose of study evaluation; (3) study information is disclosed to provide appropriate medical assistance to participants.

7. Schedules

In this study, it will take about 10 months from the preparation before initiating this study to the completion of the summary report. The schedule of clinical trial is shown as follows (for reference only):

Implementation process of clinical trial	Proposed duration
1. Preparation before clinical trial	18 days
2. IEC Review Approval	3 days
3. Recruitment and enrollment of the first participant	1 month
4. 28-day visit after the last participant completes the last dose	
5. First analysis	1 month
6. First analysis report	
7. 6-month visit after the last participant completes the last immunization	6 months
8. Final analysis	1 month
9. Summary Report	

8. Ethical Approval

8.1 Ethical Review and Approval

The PI should submit the clinical trial protocol and all necessary additional documents to the IEC for initial review:

- Clinical study protocol (indicated with version No. / date)
- Informed Consent Form (indicated with version No./date)
- Participant recruitment materials (indicated with version No. / date)
- Diary card (indicated with version No. / date)
- Contact card (indicated with version No. / date)
- Vaccination Visit Record (indicated with version No./date)
- PI's CV
- Drug clinical trial approval from the NMPA

After the above study documents are reviewed and approved by the IEC, a written approval certificate will be issued to the investigators.

8.2 Follow-up Review

Whether the methods for inclusion of participants and the information provided to participants are complete and understandable; whether the methods for obtaining informed consent are appropriate; whether SAEs are reported timely; and whether timely medical treatment can be provided for the SAE related to inoculation with the candidate vaccine.

During the whole trial, the IEC should supervise whether the risk-benefit ratio of the study is increased and whether the rights and interests of participants are effectively protected.

8.3 Potential risks and minimization of risks

8.3.1 Benefits and risks

It is expected that participants in this program will likely gain an improved level of immune response against SARS-CoV-2 from a booster dose of COVID-inactived vaccine or immune protection against influenza viruses from a booster dose of influenza vaccine. Mass vaccinations of both inactivated and influenza vaccines have demonstrated good safety, so no significant increase in safety risk is expected with an additional dose of vaccine. Participants in this clinical trial will not need to pay for the inoculation with investigational vaccines. Participants in this trial are provided with reasonable transportation fee, charge for loss of working time, blood collection compensation and nutrition fee. During participating in this clinical trial, participants will receive one dose of inactived COVID-19 vaccine or influenza vaccine. At the same time, injection of vaccines may cause some ARs. Common ARs of vaccination include: pyrexia, injection site tenderness, redness and swelling. Generally, the AEs will be alleviated within 3 - 5 days after occurrence.

8.3.2 Vaccination

Qualified inoculation consumables will be purchased, and aseptic inoculation will be performed in strict accordance with standard method to avoid AEs caused by improper inoculation or inoculation error.

If a participant experiences a grade 3 or above AR during the safety observation period, or experiences a SAE related or possibly related to the candidate vaccine, he/she should be able to receive timely medical treatment, and if necessary, the “green channel for medical treatment” should be immediately initiated for emergency treatment.

8.3.3 Blood specimen collection

After qualification review by the PI, the experienced nursing staff will be employed to collect venous blood samples after training as per the specified procedures to minimize pains or risks (including pain and less chance of venipuncture site infection) from which the participants suffered.
