# Supplementary material for: Safety and immunogenicity of heterologous boost immunization with an adenovirus type-5-vectored and protein-subunit-based COVID-19 vaccine (Convidecia/ZF2001): A randomized, observer-blinded, placebo-controlled trial
Source: PLoS Med. 2022 May 26;19(5):e1003953. doi: 10.1371/journal.pmed.1003953 (PMC9187065; doi:10.1371/journal.pmed.1003953)
Supplement: S1 Text — (DOC) [file pmed.1003953.s003.doc]

**S1 Text. The inclusion and exclusion criteria**

| **Inclusion criteria** |
| --- |
| - The subjects over 18 years old who has completed one dose of recombinant Ad5 vectored COVID-19 vaccine; - The subjects can provide with informed consent and sign informed consent form (ICF); - The subjects are able to and willing to comply with the requirements of the clinical trial program and can complete the 6-month follow-up of the study; - Axillary temperature ≤ 37.0 ℃. - Individuals who are in good health condition at the time of entry into the trial as determined by medical history, physical examination and clinical judgment of the investigators and meet the requirements of these products immunization |
| **Exclusion Criteria** |
| - Have the medical history or family history of convulsion, epilepsy, encephalopathy and psychosis; - Be allergic to any component of the research vaccines, or used to have a history of hypersensitivity or serious reactions to vaccination; - Women with positive urine pregnancy test, pregnant or breast-feeding, or have a pregnancy plan within six months; - Have acute febrile diseases and infectious diseases; - Have severe chronic diseases or condition in progress cannot be smoothly controlled, such as asthma, diabetes, thyroid disease; - Congenital or acquired angioedema / neuroedema. - Have the history of urticaria 1 year before receiving the trial vaccine. - Have asplenia or functional asplenia. - Have thrombocytopenia or other coagulation disorders (which may cause contraindications for intramuscular injection); - Have the history of immunosuppressive therapy, antiallergy therapy, cytotoxic therapy or inhaled corticosteroids (excluding corticosteroid spray therapy for allergic rhinitis, and acute corticosteroid therapy without dermatitis) over the past 6 months; - Have received blood products within 4 months before injection of trial vaccines; - Have received another investigational product within 1 month before injection of trial vaccine; - Have received attenuated vaccine within 1 month before injection of trial vaccine except the recombinant Ad5 vectored COVID-19 vaccine; - Have received subunit or inactivated vaccine within 14 days before the vaccination with trial vaccine; - Under anti tuberculosis treatment; - Not be able to follow the protocol, or not be able to understand the informed consent according to the researcher's judgment, due to various medical, psychological, social or other conditions. |
