# Supplementary material for: Safety and immunogenicity of heterologous boost immunization with an adenovirus type-5-vectored and protein-subunit-based COVID-19 vaccine (Convidecia/ZF2001): A randomized, observer-blinded, placebo-controlled trial
Source: PLoS Med. 2022 May 26;19(5):e1003953. doi: 10.1371/journal.pmed.1003953 (PMC9187065; doi:10.1371/journal.pmed.1003953)
Supplement: S2 Text — (DOC) [file pmed.1003953.s004.doc]

**S2 Text. Immunogenicity assay**

***Microneutralization assay.*** SARS-CoV-2-specific neutralizing antibody titer in serum was determined using a cytopathic effect (CPE)-based microneutralization assay with the SARS-CoV-2 virus strain in Vero-E6 cells (wild-type SARS-CoV-2: BetaCoV/Jiangsu/JS02/2020 (EPI_ISL_411952); delta variant B.1.617.2: hCoV-19/China/JS07/2021(EPI_ISL_4515846); omicrom variant BA.1.1: hCoV-19/Jiangsu/JS01/2022(EPI_ISL_12511653)). Heat-inactivated sera were two-fold serially diluted, and then mixed with the same volume of viral solution to achieve a final concentration of 50% tissue culture infectious dose of 100 in each well (100 TCID50). The serum dilution for microneutralization assay started from 1:8 to 1:256 for wild-type, and 1:4 to 1:128 for delta variant B.1.617.2, and 1:4 to 1:128 for omicron variant BA.1.1. The reported titer was the reciprocal of the highest sample dilution that protected at least 50% of cells from cytopathic effects. If no neutralization reaction was observed at the initial serum dilution, half of the limit of quantification was calculated.

***Binding-antibody assays.*** Binding antibodies against receptor-binding domain (RBD) and spike protein were detected by an indirect ELISA assay with a cutoff titer of 1:10. The commercial ELISA kits (Vazyme Biotech Co.,Ltd) were used for the detection. Briefly, serum samples were serially diluted (anti-RBD antibody detection, 1:10 to 1:1280; anti-Spike antibody detection, 1:10 to 1:21870) with sample diluent and tested in 96-well plates costed with a recombinant RBD or Spike antigen. IgG was detected using an anti-human IgG monoclonal antibody conjugated to horseradish peroxidase (HRP) diluted for each ELISA assay and TMB substrate. Data collection was performed using a Multiskan GO reader (Thermo Fisher) to detect optical density (OD) at 450 and 630 nm using SkanIt Software for Microplate Readers (version 4.1.0.43). A monoclonal antibody with neutralizing activity specific to the SARS-CoV-2 RBD or Spike protein was used as a calibrator, which was used to generate a standard curve to convert OD units into relative units per milliliter (RU/ml) in the ELISA.

***The calibration and harmonization of WHO international standard.*** The WHO international standard for anti-SARS-CoV-2 immunoglobulin (NIBSC code 20/136) was used side by side as reference with the serum samples measured in this study for calibration and harmonization of the serological assays. The WHO reference serum 1000 IU/mL equivalent to live viral neutralizing antibody titer of 1:320 against the wild-type and a titer of 1:80 against the Delta variant B.1.617.2, while the WHO reference (1000 IU/mL) is equivalent to binding antibody concentrations of 1000 RU/ml. Seroconversion was defined as at least a fourfold increase in the antibody titers at different time points after boost immunization compared to baseline level (at 28 days post prime dose). Seropositive was defined as a titer ≥1:8 for neutralizing antibodies to the wild-type virus, and ≥1:4 for neutralizing antibodies to the Delta variant and ≥1:4 for neutralizing antibodies to the Omicron variant.

***Enzyme-linked immunospot (ELISpot) assay.*** The cellular immune responses of the expression of interferon (IFN) γ stimulated by the overlapping peptide pool of spike glycoprotein were detected by ELISpot assay (Mabtech, Stockholm, Sweden). PBMCs were isolated by Ficoll-Paque PLUS (Cytiva) density gradient centrifugation and cryopreserved before analysis. Per well, 100,000 isolated PBMCs were stimulated with peptide pools covering the full-length spike glycoprotein at a concentration of 1μg in the plates. Plates were scanned, and spots were counted on the Cellular Technology ImmunoSpot Analyzer (AID Diagnostika GmbH) for AID EliSpot 7.0 software. IFN γ-secreting spots forming cells was calculated as the number of spots forming cells in the presence of peptides minus the number of that without peptides, and were multiplied by ten to express frequencies pre 10^6^ PBMCs.
