# Supplementary material for: Safety and immunogenicity of heterologous boost immunization with an adenovirus type-5-vectored and protein-subunit-based COVID-19 vaccine (Convidecia/ZF2001): A randomized, observer-blinded, placebo-controlled trial
Source: PLoS Med. 2022 May 26;19(5):e1003953. doi: 10.1371/journal.pmed.1003953 (PMC9187065; doi:10.1371/journal.pmed.1003953)
Supplement: S1 Data — Table A. Baseline characteristics of the participants from external comparators. Table B. Adverse reactions occurred within 7 days and unsolicited adverse events within 28 days post first boost. Table C. Live virus neutralizing antibodies after prime and boost dose. Table D. Wild-type virus neutralizing antibodies after prime and boost dose according to age. Table E. SARS-CoV-2-specific anti-RBD IgG and anti-S IgG antibodies after prime and boost dose. (DOCX) [file pmed.1003953.s005.docx]

# Table A. Baseline characteristics of the participants from external comparators

|  | **CV/CV (D0-D56)**  **regimen** | **CV/CV (D0-M6)**  **regimen** |
| --- | --- | --- |
| N | 40 | 20 |
| Age, years | 59.0 (10.9) | 40.2 (10.9) |
| Age group | | |
| 18-59 years | 18 (45%) | 20 (100%) |
| ≥ 60 years | 22 (55%) | NA |
| Sex | | |
| Female | 22 (55%) | 11（55%） |
| Male | 18 (45%) | 9（45%） |
| Body-mass index(kg/m^2^) | 25.8 (3.5) | 23.3 (2.2) |
| Underlying diseases | | |
| Yes | 2 (5%) | 0 |
| No | 38 (95%) | 0 |

Data are number of participants (%) or mean (SD). CV/CV (D0-D56)=receiving Convidecia/Convidecia at day 0 and day 56; CV/CV (D0-M6)=receiving Convidecia/Convidecia at day 0 and month 6.

# Table B. Adverse reactions occurred within 7 days and unsolicited adverse events within 28 days post 1st boost.

|  | **0-28 day regimen** | | | | |  | **0-56 day regimen** | | |  |
| --- | --- | --- | --- | --- | --- | --- | --- | --- | --- | --- |
|  | **Vaccine group**  **(N=40)** | | **Placebo group**  **(N=20)** | | ***P* value** |  | **Vaccine group (N=40)** | | **Placebo group (N=20)** | ***P* value** |
| **Adverse reaction within 7 days post vaccination** | | | | | | | | | | |
| Total | 5 (12.5) | | 2 (10) | | ＞0.999 |  | 1 (2.5) | | 0 (0.0) | ＞0.999 |
| **Injection-site adverse reaction within 7 days post vaccination** | | | | | | | | | | |
| Total | 5 (12.5) | | 2(10.0) | | ＞0.999 |  | 1 (2.5) | | 0 (0.0) | ＞0.999 |
| Pain | 5 (12.5) | | 1 (5.0) | | 0.648 |  | 1 (2.5) | | 0 (0.0) | ＞0.999 |
| Redness | 0 (0.0) | | 1 (5.0) | | 0.721 |  | 0 (0.0) | | 0 (0.0) | / |
| Swelling | 0 (0.0) | | 0 (0.0) | | / |  | 0 (0.0) | | 0 (0.0) | / |
| Induration | 1 (2.5) | | 0 (0.0) | | ＞0.999 |  | 0 (0.0) | | 0 (0.0) | / |
| **Systemic adverse reaction within 7 days post vaccination** | | | | | | | | | | |
| Total | 0 (0.0) | 0 (0.0) | | / | |  | 0 (0.0) | 0 (0.0) | | / |
| **Unsolicited adverse event within 28 days post vaccination** | | | | | | | | | | |
| Total | 0 (0.0) | 1 (5.0) | | 0.721 | |  | 1 (2.5) | 2 (10.0) | | 0.529 |

Data are n (%): n refers to the number of participants, % refers to the proportion of participants; N refers to the number of participants included in the safety analysis; All the reported adverse reactions were mild. 0-28 day regimen=receiving either one dose of ZF2001 (vaccine) or TIV (placebo) 28 days post priming with Convidecia; 0-56 day regimen=receiving either one dose of ZF2001 (vaccine) or TIV (placebo) 56 days post priming with Convidecia. TIV= Trivalent inactivated influenza vaccine.

# Table C. Live virus neutralizing antibodies after post prime and boost dose.

|  | **CV/ZF/ZF**  **(D0-D28-M5)**  **regimen** | **CV/ZF**  **(D0-M5)**  **regimen** | ***P* value** |  | **CV/ZF/ZF**  **(D0-D56-M6)**  **regimen** | **CV/ZF**  **(D0-M6)**  **regimen** | ***P* value** |
| --- | --- | --- | --- | --- | --- | --- | --- |
| **Live virus neutralizing antibodies against wild-type isolate** | | | | | | | |
| **Day 28 after priming** | | | | | | | |
| N | 40 | 20 |  |  | 40 | 20 |  |
| GMT | 7.6  (5.8,10.0) | 8.6  (5.4, 13.6) | 0.626 |  | 8.1  (6.1, 10.8) | 9.9  (6.4, 15.2) | 0.446 |
| GMT(IU/mL)* | 23.7  (18.0, 31.3) | 26.8  (17.0, 42.3) | 0.626 |  | 25.4  (19.1, 33.9) | 30.8  (19.9,47.6) | 0.446 |
| Seropositive rate(%) | 50.0  (33.8, 66.2) | 45.0  (23.1, 68.5) | 0.715 |  | 50.0  (33.8, 66.2) | 60.0  (36.1, 80.9) | 0.464 |
| **Day 56 after priming** | | | | | | | |
| N | NA | NA | NA |  | 40 | 20 |  |
| GMT | NA | NA | NA |  | 6.5  (4.9, 8.5) | 8.3  (4.9, 13.9) | 0.347 |
| GMT (IU/mL)* | NA | NA | NA |  | 20.3  (15.6, 26.5) | 25.9  (15.4, 43.6) | 0.347 |
| GMFI | NA | NA | NA |  | 0.8  (0.6, 1.0) | 0.8  (0.6, 1.2) | 0.793 |
| Seroconversion rate(%) | NA | NA | NA |  | 2.5  (0.1, 13.2) | 5.0  (0.1, 24.9) | 1.0000 |
| Seropositive rate(%) | NA | NA | NA |  | 30.0  (16.6, 46.5) | 45.0  (23.1, 68.5) | 0.251 |
| **Day 14 post-1st boosting** | | | | | | | |
| N | 40 | 20 |  |  | 39 | 20 |  |
| GMT | 18.7  (13.7, 25.5) | 9.2  (5.6, 15.2) | 0.013 |  | 25.9  (17.0, 39.3) | 7.7  (5.0, 11.9) | <0.001 |
| GMT (IU/mL)* | 58.4  (42.8, 79.8) | 28.7  (17.4, 47.4) | 0.013 |  | 80.8  (53.1, 122.9) | 24.2  (15.8, 37.0) | <0.001 |
| GMFI | 2.5  (1.9, 3.1) | 1.1  (0.9, 1.4) | <0.001 |  | 3.3  (2.4, 4.4) | 0.8  (0.6, 1.0) | <0.001 |
| Seroconversion rate(%) | 45.0  (29.3, 61.5) | 5.0  (0.1, 24.9 ) | 0.002 |  | 51.3  (34.8, 67.6) | 0.00(-) | <0.001 |
| Seropositive rate(%) | 85.0  (70.2, 94.3) | 55.0  (33.2, 76.8) | 0.013 |  | 85.0  (70.2, 94.3) | 45.0  (23.1, 68.5) | <0.001 |
| **Day 28 post-1st boosting** | | | | | | | |
| N | 40 | 20 |  |  | 39 | 20 |  |
| GMT | 11.9  (8.9, 15.9) | 6.1  (4.4, 8.4) | 0.005 |  | 22.0  (14.7, 33.1) | 6.9  (4.2, 11.5) | <0.001 |
| GMT (IU/mL)* | 37.3  (27.9, 49.7) | 18.9  (13.7, 26.2) | 0.005 |  | 68.8  (45.9, 103.3) | 21.8  (13.2, 35.9) | <0.001 |
| GMFI | 1.6  (1.3, 1.9) | 0.7  (0.5, 0.9) | <0.001 |  | 2.8  (2.1, 3.7) | 0.7  (0.5, 1.0) | <0.001 |
| Seroconversion rate(%) | 12.5  (4.2, 26.8) | 0.00(-) | 0.159 |  | 56.4  (39.6, 72.2) | 5.0  (0.1, 24.9) | <0.001 |
| Seropositive rate(%) | 80.0  (64.3, 90.9) | 35.0  (15.4,59.2) | <0.001 |  | 77.5  (61.6, 89.2) | 35.0  (15.4, 59.2) | <0.001 |
| **Day 14 post-2nd boosting** | | | | | | | |
| N | 40 | 19 |  |  | 36 | 18 |  |
| GMT | 107.2  (73.7, 155.8) | 90.5  (45.6, 179.8) | 0.629 |  | 141.2  (83.4, 238.8) | 94.1  (44.0,200.9) | 0.367 |
| GMT (IU/mL)* | 334.9  (230.3, 486.9) | 282.8  (142.4, 561.8) | 0.629 |  | 441.2  (260.8, 746.4) | 293.9  (137.6,627.9) | 0.367 |
| GMFI | 14.1  (9.5, 20.9) | 10.7  (5.8, 19.8) | 0.427 |  | 17.3  (11.1, 26.9) | 8.9  (4.8, 16.7) | 0.084 |
| Seroconversion rate (%) | 90.0  (75.8, 97.1) | 89.5  (66.9, 98.7) | 1.000 |  | 91.7  (77.5, 98.3) | 83.3  (58.6, 96.4) | 0.388 |
| Seropositive rate(%) | 100.0  (90.9, 100.0) | 94.7  (73.9, 99.9) | 0.328 |  | 94.4  (81.3, 99.3) | 94.4  (72.2, 99.9) | 1.000 |
| **Live virus neutralizing antibodies against Delta variant B.1.617.2** | | | | | | | |
| **Day 28 after priming** | | | | | | | |
| N | 40 | 20 |  |  | 40 | 20 |  |
| GMT | 2.6  (2.2, 3.2) | 2.9  (2.1, 4.2) | 0.539 |  | 2.9  (2.3, 3.7) | 2.8  (2.0, 3.9) | 0.861 |
| Seropositive rate(%) | 25.0  (12.7, 41.2) | 27.8  (9.7, 53.5) | 1.000 |  | 25.0  (12.7, 41.2) | 25.0  (8.7, 49.1) | 1.000 |
| Wild-type to Delta ratio | 2.9  (2.4,3.4) | 2.7  (2.0, 3.7) | 0.746 |  | 2.8  (2.3,3.3) | 3.4  (2.6,4.8) | 0.174 |
| **Day 14 post-2nd boosting** | | | | | | | |
| N | 40 | 19 |  |  | 36 | 18 |  |
| GMT | 38.0  (26.7, 54.2) | 29.7  (15.3, 57.9) | 0.465 |  | 41.9  (27.0, 65.0) | 34.6  (18.1, 65.9) | 0.609 |
| GMFI | 14.4  (10.1, 20.6) | 10.6  (5.6, 20.3) | 0.368 |  | 14.3  (9.5, 21.3) | 11.8  (6.3, 21.8) | 0.583 |
| Seropositive rate(%) | 97.5  (86.8, 99.9) | 89.5  (66.9, 98.7) | 0.240 |  | 91.7  (77.5, 98.3) | 94.4  (72.7, 99.9) | 1.000 |
| Wild-type to Delta ratio | 2.9  (2.1,3.7) | 3.1  (2.2,4.3) | 0.699 |  | 3.4  (2.9,4.2) | 2.9  (2.1,3.8) | 0.204 |

Data shown are geometric mean (95% CI) for continuous variables, and the percent (95%CI) for binary variables. *Neutralizing antibody (IU/ml) was converted to the WHO international standard (NIBSC code 20/136) using the following conversion factors: IU/ml for wild-type isolate=100 TCID_50_ ×3.125. Seroconversion was defined as at least a fourfold increase in the antibody titers at different time points after boost immunisation compared to baseline level (at 28 days post prime dose). Seropositive was defined as a titer ≥1:8 for neutralizing antibodies to the wild-type virus and ≥1:4 for neutralizing antibodies to the Delta variant. IU/ml=International units per milliliter, TCID_50_=50% tissue culture infectious dose. GMT=geometric mean titer; GMFI=geometric mean fold increase. NA=Not Applicable. CV/ZF/ZF (D0-D28-M5)=receiving Convidecia/ZF2001/ZF2001 at day 0, day 28 and month 5; CV/ZF (D0-M5)=receiving Convidecia/ZF2001 at day 0 and month 5; CV/ZF/ZF (D0-D56-M6)=receiving Convidecia/ZF2001/ZF2001 at day 0, day 56 and month 6; CV/ZF (D0-M6)=receiving Convidecia/ZF2001 at day 0 and month 6.

.

# Table D. Wild-type virus neutralizing antibodies after prime and boost dose accoding to age.

|  | **N** | **CV/ZF/ZF**  **(D0-D28-M5)**  **regimen** | **N** | **CV/ZF**  **(D0-M5)**  **regimen** | ***P* value** | **N** | **CV/ZF/ZF**  **(D0-D6-M6)**  **regimen** | **N** | **CV/ZF**  **(D0-M6)**  **regimen** | ***P* value** |
| --- | --- | --- | --- | --- | --- | --- | --- | --- | --- | --- |
| **Day 28 after priming** | | | | | | | | | | |
| 18-59 years | 20 | 7.5  (5.3,10.6) | 10 | 9.2  (5.2,16.1) | 0.484 | 20 | 10.2  (6.2,16.8) | 10 | 11.3 (6.0,21.2) | 0.792 |
| ≥ 60 years | 20 | 7.7  (4.9,12.3) | 10 | 8.0  (3.4,18.6) | 0.933 | 20 | 6.5  (4.8,8.8) | 10 | 8.6  (4.2,17.6) | 0.364 |
| **Day 56 after priming** | | | | | | | | | | |
| 18-59 years | NA | NA | NA | NA | NA | 20 | 6.5  (4.4,9.7) | 10 | 9.2  (3.9,21.2) | 0.359 |
| ≥ 60 years | NA | NA | NA | NA | NA | 20 | 6.5  (4.4,9.7) | 10 | 7.5  (3.4,16.5) | 0.705 |
| **Day 14 post-1st boosting** | | | | | | | | | | |
| 18-59 years | 20 | 19.7 (13.3,29.3) | 10 | 9.2  (4.8,17.7) | 0.031 | 19 | 34.4 (18.4,64.6) | 10 | 8.6  (4.5,16.2) | 0.006 |
| ≥ 60 years | 20 | 17.8 (10.6,29.8) | 10 | 9.2  (3.7,22.6) | 0.153 | 20 | 19.7 (10.9,35.4) | 10 | 6.9  (3.5,13.9) | 0.029 |
| **Day 28 post-1st boosting** | | | | | | | | | | |
| 18-59 years | 20 | 11.7 (8.2,16.7) | 10 | 6.1  (3.8,9.8) | 0.028 | 19 | 27.7 (14.8,51.7) | 10 | 6.9  (3.2,15.0) | 0.008 |
| ≥ 60 years | 20 | 12.1 (7.5,19.7) | 10 | 6.1  (3.6,10.3) | 0.072 | 20 | 17.8 (10.1,31.1) | 10 | 6.9  (3.1,15.6) | 0.049 |
| **Day 14 post-2st boosting** | | | | | | | | | | |
| 18-59 years | 20 | 128.0  (73.4,223.4) | 9 | 103.9  (30.2,358.5) | 0.702 | 19 | 205.7 (135.5,312.2) | 10 | 157.6 (56.8,437.1) | 0.535 |
| ≥ 60 years | 20 | 90.5 (52.7,155.6) | 10 | 78.8 (32.8,189.2) | 0.764 | 17 | 92.7 (32.8,262.0) | 8 | 49.4 (14.3,169.9) | 0.440 |

Data presented are geometric mean (95% CI) of neutralizing antibodies to wild-type SARS-CoV-2 28 days after prime dose, 14 days after 1st and 2nd boost dose according to age (18-59 years and ≥60 years). NA=Not Applicable. CV/ZF/ZF (D0-D28-M5)=receiving Convidecia/ZF2001/ZF2001 at day 0, day 28 and month 5; CV/ZF (D0-M5)=receiving Convidecia/ZF2001 at day 0 and month 5; CV/ZF/ZF (D0-D56-M6)=receiving Convidecia/ZF2001/ZF2001 at day 0, day 56 and month 6; CV/ZF (D0-M6)=receiving Convidecia/ZF2001 at day 0 and month 6.

# Table E. SARS-CoV-2-specific anti-RBD IgG and anti-S IgG antibodies after prime and boost dose.

|  | **CV/ZF/ZF**  **(D0-D28-M5)**  **regimen** | **CV/ZF**  **(D0-M5)**  **regimen** | ***P* value** |  | **CV/ZF/ZF**  **(D0-D56-M6)**  **regimen** | **CV/ZF**  **(D0-M6)**  **regimen** | ***P* value** |
| --- | --- | --- | --- | --- | --- | --- | --- |
| **SARS-CoV-2 anti RBD IgG** | | | | | | | |
| **Day 28 after priming** | | | | | | | |
| N | 40 | 20 |  |  | 40 | 20 |  |
| GMC | 43.7  (30.3, 62.9) | 53.0  (27.8, 100.9) | 0.567 |  | 49.0  (31.5, 76.3) | 60.0  (38.5, 93.5) | 0.545 |
| **Day 56 after priming** | | | | | | | |
| N | NA | NA | NA |  | 40 | 20 |  |
| GMC | NA | NA | NA |  | 37.3  (22.7, 61.3) | 43.9  (24.8, 77.8) | 0.673 |
| GMFI | NA | NA | NA |  | 0.8  (0.7, 0.9) | 0.7  (0.6, 0.9) | 0.554 |
| Seroconversion rate(%) | NA | NA | NA |  | 3.1(0.1, 15.8) | 0.00(-) | 1.000 |
| **Day 14 post-1st boosting** | | | | | | | |
| N | 40 | 20 |  |  | 39 | 20 |  |
| GMC | 258.8  (176.8,278.7) | 50.0  (26.1, 95.8) | <0.001 |  | 335.8  (205.9, 547.6) | 36.0  (20.7, 62.6) | <0.001 |
| GMFI | 6.2  (4.8, 7.9) | 0.9  (0.8, 1.2) | <0.001 |  | 8.9  (5.5, 14.7) | 0.6  (0.5, 0.8) | <0.001 |
| Seroconversion rate(%) | 63.2  (45.9, 78.2) | 0.00(-) | <0.001 |  | 75.8  (57.7, 88.9) | 0.00(-) | <0.001 |
| **Day 28 post-1st boosting** | | | | | | | |
| N | 40 | 20 |  |  | 39 | 20 |  |
| GMC | 210.1  (146.3, 301.8) | 42.0  (22.5, 78.4) | <0.001 |  | 265.7  (167.0, 422.7) | 29.0  (16.8, 50.2) | <0.001 |
| GMFI | 5.0  (3.9, 6.4) | 0.7  (0.5, 0.9) | <0.001 |  | 7.0  (4.5, 11.1) | 0.5  (0.4, 0.7) | <0.001 |
| Seroconversion rate(%) | 60.5  (43.4, 75.9) | 0.00(-) | <0.001 |  | 63.6  (45.1, 79.6) | 0.00(-) | <0.001 |
| **Day 14 post-2nd boosting** | | | | | | | |
| N | 40 | 19 |  |  | 36 | 18 |  |
| GMC | 695.6  (465.9,1038.5) | 514.7  (255.9,1035.2) | 0.416 |  | 951.4  (594.0, 1523.9) | 534.5  (256.7,1112.9) | 0.165 |
| GMFI | 18.2  (12.5, 26.6) | 11.5  (7.9, 16.8) | 0.139 |  | 27.8  (17.4, 44.4) | 11.3  (6.6, 19.3) | 0.016 |
| Seroconversion rate(%) | 86.8  (71.9, 95.6) | 94.1  (71.3, 99.9) | 0.654 |  | 90.0  (73.5, 97.9) | 88.2  (63.6, 98.5) | 1.000 |
| **SARS-CoV-2 anti-S IgG** | | | | | | | |
| **Day 28 after priming** | | | | | | | |
| N | 40 | 20 |  |  | 40 | 20 |  |
| GMC | 57.8  (39.8, 83.9) | 54.6  (28.0, 106.3) | 0.869 |  | 64.1  (39.3, 104.7) | 83.8  (46.9, 149.8) | 0.498 |
| **Day 56 after priming** | | | | | | | |
| N | NA | NA | NA |  | 40 | 20 |  |
| GMC | NA | NA | NA |  | 35.9  (21.4, 60.2) | 56.9  (32.5, 99.4) | 0.265 |
| GMFI | NA | NA | NA |  | 0.6  (0.5, 0.8) | 0.7  (0.5, 0.9) | 0.582 |
| Seroconversion rate(%) | NA | NA | NA |  | 5.1  (0.6, 17.3) | 0.00(-) | 0.544 |
| **Day 14 post-1st boosting** | | | | | | | |
| N | 40 | 20 |  |  | 39 | 20 |  |
| GMC | 196.6  (128.3,301.1) | 49.3  (24.8, 97.7) | <0.001 |  | 222.3  (130.9, 377.5) | 47.7  (26.1, 87.1) | <0.001 |
| GMFI | 3.8  (2.9, 4.8) | 0.9  (0.7, 1.1) | <0.001 |  | 4.0  (2.8, 5.8) | 0.6  (0.4, 0.8) | <0.001 |
| Seroconversion rate(%) | 43.6  (27.8, 60.4) | 0.00(-) | <0.001 |  | 44.7  (28.6, 61.7) | 0.00(-) | <0.001 |
| **Day 28 post-1st boosting** | | | | | | | |
| N | 40 | 20 |  |  | 39 | 20 |  |
| GMC | 163.2  (108.9,244.5) | 37.7  (18.7,75.9) | <0.001 |  | 198.9  (123.7, 320.1) | 34.3  (18.9, 62.0) | <0.001 |
| GMFI | 3.1  (2.5, 3.9) | 0.7  (0.6, 0.9) | <0.001 |  | 3.6  (2.6, 4.9) | 0.4  (0.3, 0.6) | <0.001 |
| Seroconversion rate(%) | 30.8  (17.0, 47.6) | 0.00(-) | 0.005 |  | 44.7  (28.6, 61.7) | 0.00(-) | <0.001 |
| **Day 14 post-2nd boosting** | | | | | | | |
| N | 40 | 19 |  |  | 36 | 18 |  |
| GMC | 571.9  (396.9,823.9) | 412.9  (202.1,843.9) | 0.358 |  | 686.1  (435.8, 1080.4) | 407.3  (211.4, 784.9) | 0.182 |
| GMFI | 10.8  (7.3, 15.9) | 8.3  (4.7, 14.6) | 0.436 |  | 11.9  (8.0, 17.7) | 5.1(3.6, 7.2) | 0.006 |
| Seroconversion rate(%) | 79.5  (63.5, 90.7) | 78.9  (54.4, 93.9) | 1.000 |  | 85.7  (69.7, 95.2) | 61.1  (35.8, 82.7) | 0.079 |

Data shown are geometric mean (95% CI) for continuous variables, and the percent (95%CI) for binary variables. Seroconversion was defined as at least a fourfold increase in the antibody titers at different time points after boost immunisation compared to baseline level (at 28 days post prime dose). IU/ml=International units per milliliter. GMC=geometric mean concentration. GMFI=geometric mean fold increase. NA=Not Applicable. CV/ZF/ZF (D0-D28-M5)=receiving Convidecia/ZF2001/ZF2001 at of day 0, day 28 and month 5; CV/ZF (D0-M5)=receiving Convidecia/ZF2001 at day 0 and month 5; CV/ZF/ZF (D0-D56-M6)=receiving Convidecia/ZF2001/ZF2001 at day 0, day 56 and month 6; CV/ZF (D0-M6)=receiving Convidecia/ZF2001 at day 0 and month 6.
