# Supplementary figures and images for: Safety and immunogenicity of heterologous boost immunization with an adenovirus type-5-vectored and protein-subunit-based COVID-19 vaccine (Convidecia/ZF2001): A randomized, observer-blinded, placebo-controlled trial
Source: PLoS Med. 2022 May 26;19(5):e1003953. doi: 10.1371/journal.pmed.1003953 (PMC9187065; doi:10.1371/journal.pmed.1003953)

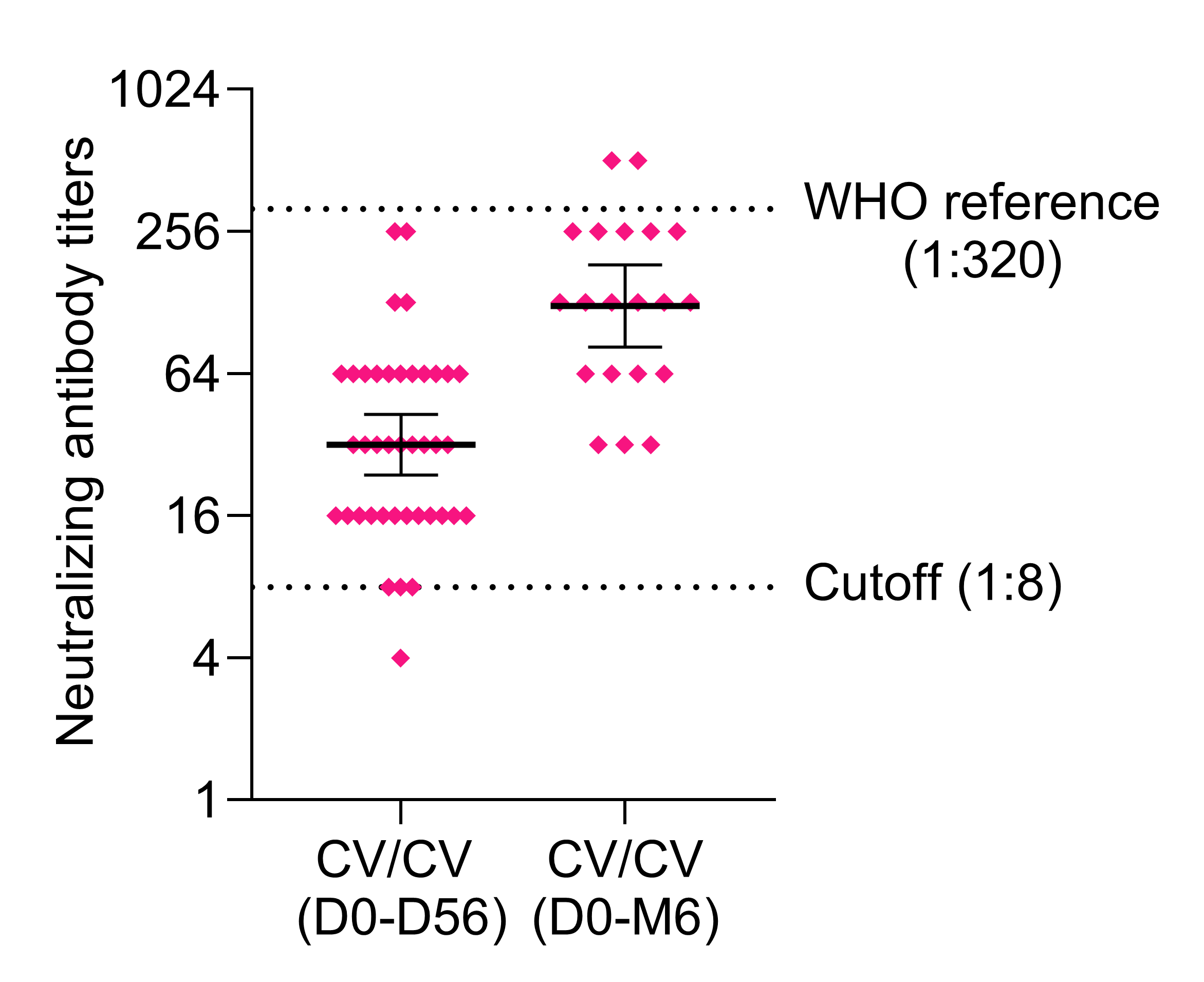

Supplement: S1 Fig — Horizontal bars show geometric mean and error bars show 95% CI. The WHO reference (1,000 IU/ ml) is equivalent to a neutralizing antibody titer of 1:320 against wild-type SARS-CoV-2. Cutoff (1:8) refers to the detection limit. CV/CV (D0-D56) = receiving Convidecia/Convidecia at day 0 and day 56. CV/CV (D0-M6) = receiving Convidecia/Convidecia at day 0 and month 6. CI, confidence interval; SARS-CoV-2, Severe Acute Respiratory Syndrome Coronavirus 2; WHO, World Health Organization. (TIF) [file pmed.1003953.s006.tif]

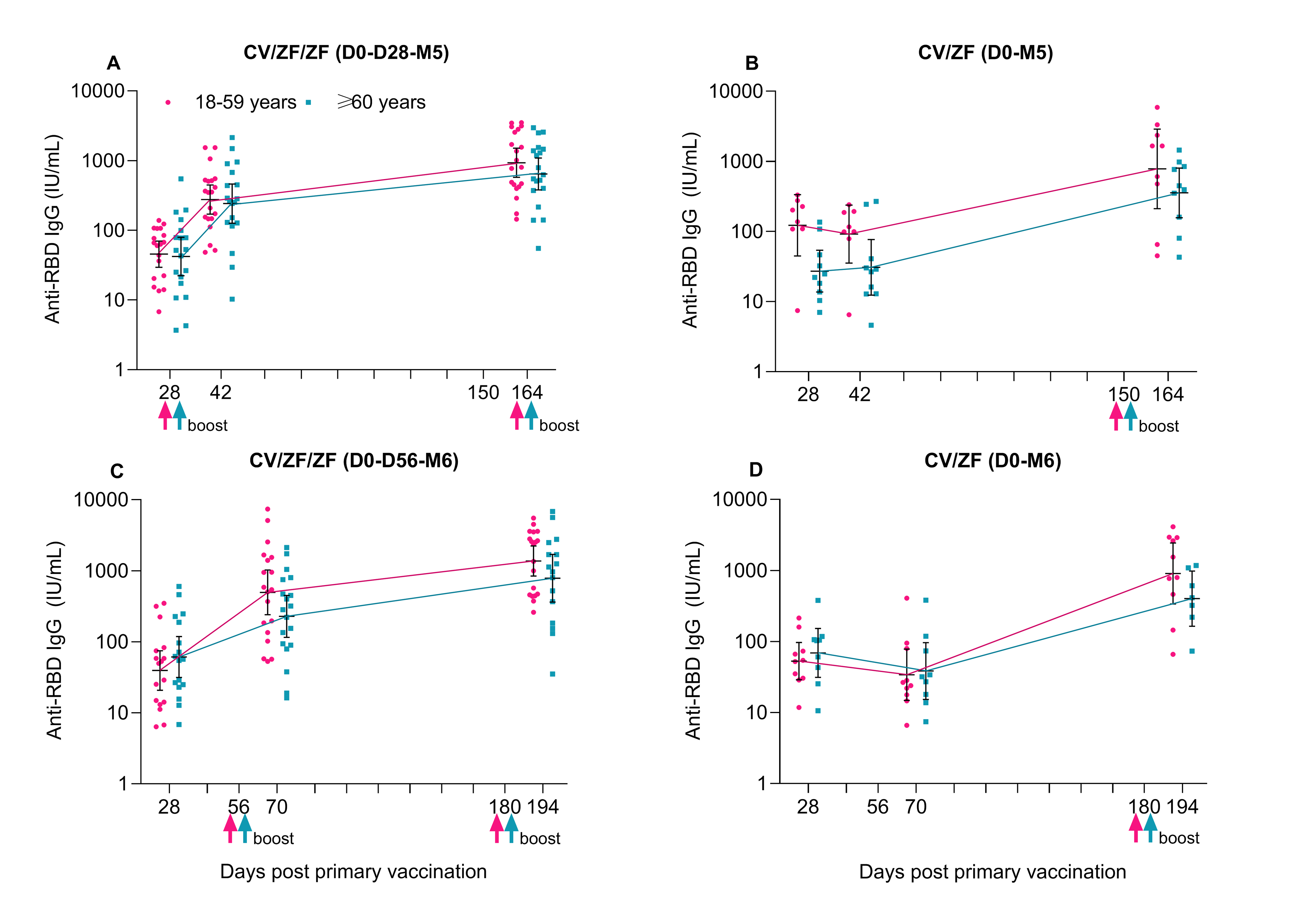

Supplement: S2 Fig — Data presented are SARS-CoV-2-specific anti-RBD IgG antibodies 28 days after prime dose, 14 days after first and second boost dose according to age (18–59 years and ≥60 years) in each regimen. Horizontal bars show geometric mean titer and error bars show 95% CI. Up arrow represents the boost immunization. RBD = SARS-CoV-2 receptor-binding domain. IU/ml = International units per milliliter. CV/ZF/ZF (D0-D28-M5) = receiving Convidecia/ZF2001/ZF2001 at day 0, day 28, and month 5; CV/ZF (D0-M5) = receiving Convidecia/ZF2001 at day 0 and month 5; CV/ZF/ZF (D0-D56-M6) = receiving Convidecia/ZF2001/ZF2001 at day 0, day 56, and month 6; CV/ZF (D0-M6) = receiving Convidecia/ZF2001 at day 0 and month 6. CI, confidence interval; IgG, immunoglobulin G; RBD, receptor-binding domain; SARS-CoV-2, Severe Acute Respiratory Syndrome Coronavirus 2. (TIF) [file pmed.1003953.s007.tif]

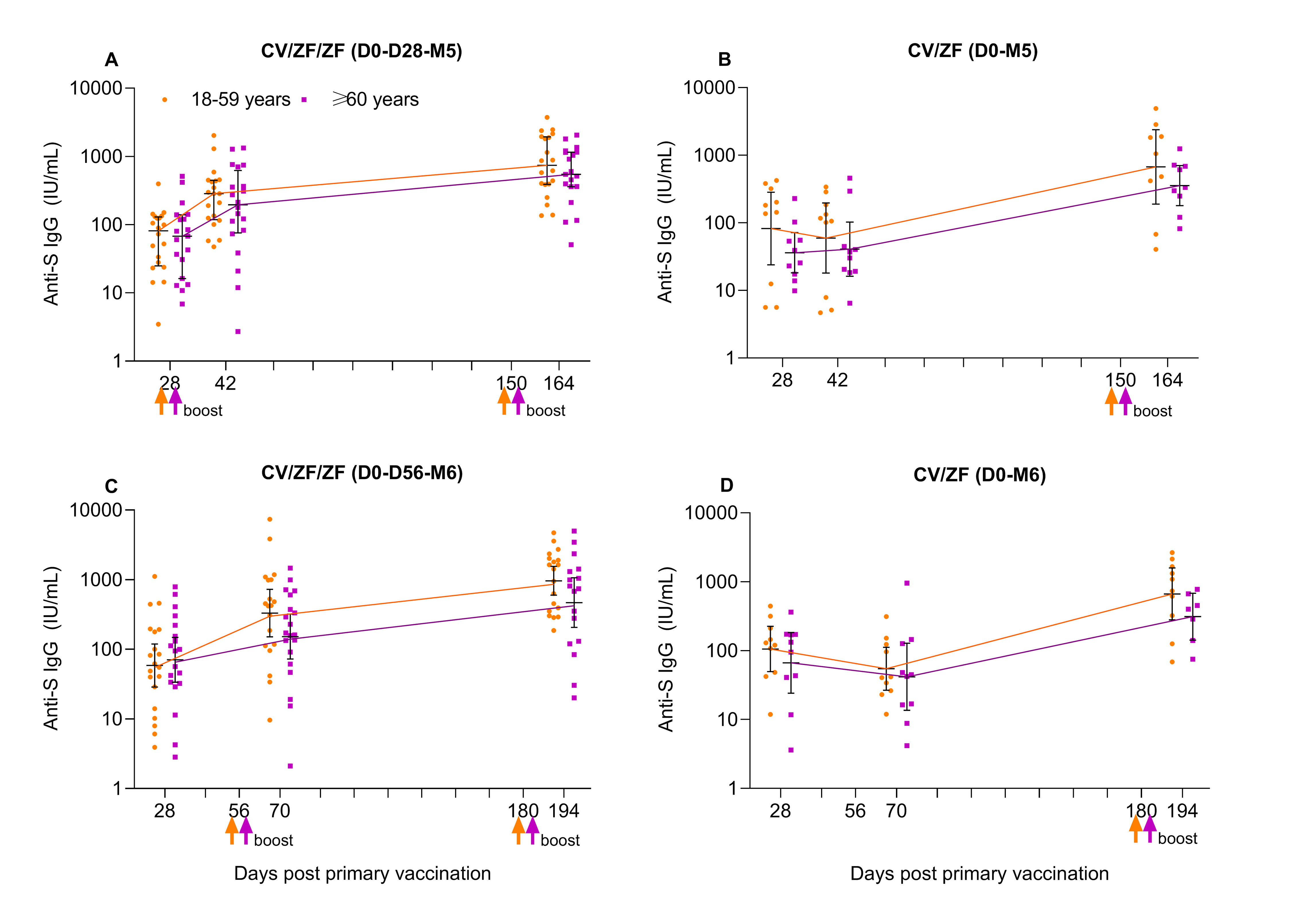

Supplement: S3 Fig — Data presented are SARS-CoV-2-specific anti-spike IgG antibodies 28 days after prime dose, 14 days after first and second boost dose according to age (18–59 years and ≥60 years) in each regimen. Horizontal bars show geometric mean titer and error bars show 95% CI. Up arrow represents the boost immunization. S = SARS-CoV-2 spike protein. IU/ml = International units per milliliter. CV/ZF/ZF (D0-D28-M5) = receiving Convidecia/ZF2001/ZF2001 at day 0, day 28, and month 5; CV/ZF (D0-M5) = receiving Convidecia/ZF2001 at day 0 and month 5; CV/ZF/ZF (D0-D56-M6) = receiving Convidecia/ZF2001/ZF2001 at day 0, day 56, and month 6; CV/ZF (D0-M6) = receiving Convidecia/ZF2001 at day 0 and month 6. CI, confidence interval; IgG, immunoglobulin G; SARS-CoV-2, Severe Acute Respiratory Syndrome Coronavirus 2. (TIF) [file pmed.1003953.s008.tif]

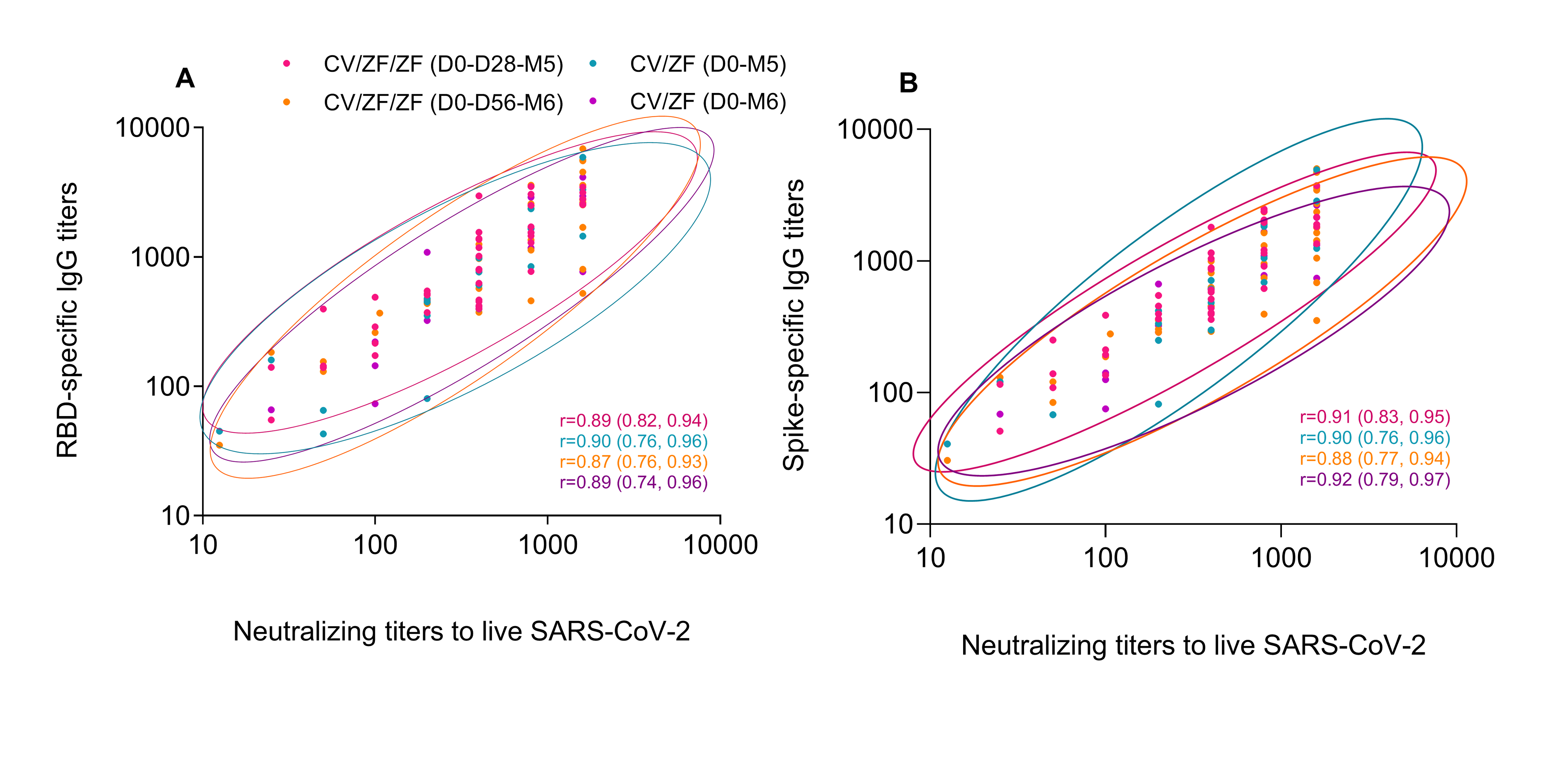

Supplement: S4 Fig — Correlations at 14 days post second boost were analyzed between neutralizing antibodies to wide-type SARS-CoV-2 and RBD-specific IgG (A), between neutralizing antibodies to wide-type SARS-CoV-2 and spike-specific IgG (B). Ellipses show the 95% CIs for different vaccine schedules, assuming multivariate normal distributions. Pearson correlation coefficients (95% CIs) are presented for each regimen. CV/ZF/ZF (D0-D28-M5) = receiving Convidecia/ZF2001/ZF2001 at day 0, day 28, and month 5; CV/ZF (D0-M5) = receiving Convidecia/ZF2001 at day 0 and month 5; CV/ZF/ZF (D0-D56-M6) = receiving Convidecia/ZF2001/ZF2001 at day 0, day 56, and month 6; CV/ZF (D0-M6) = receiving Convidecia/ZF2001 at day 0 and month 6. CI, confidence interval; IgG, immunoglobulin G; RBD, receptor-binding domain; SARS-CoV-2, Severe Acute Respiratory Syndrome Coronavirus 2. (TIF) [file pmed.1003953.s009.tif]

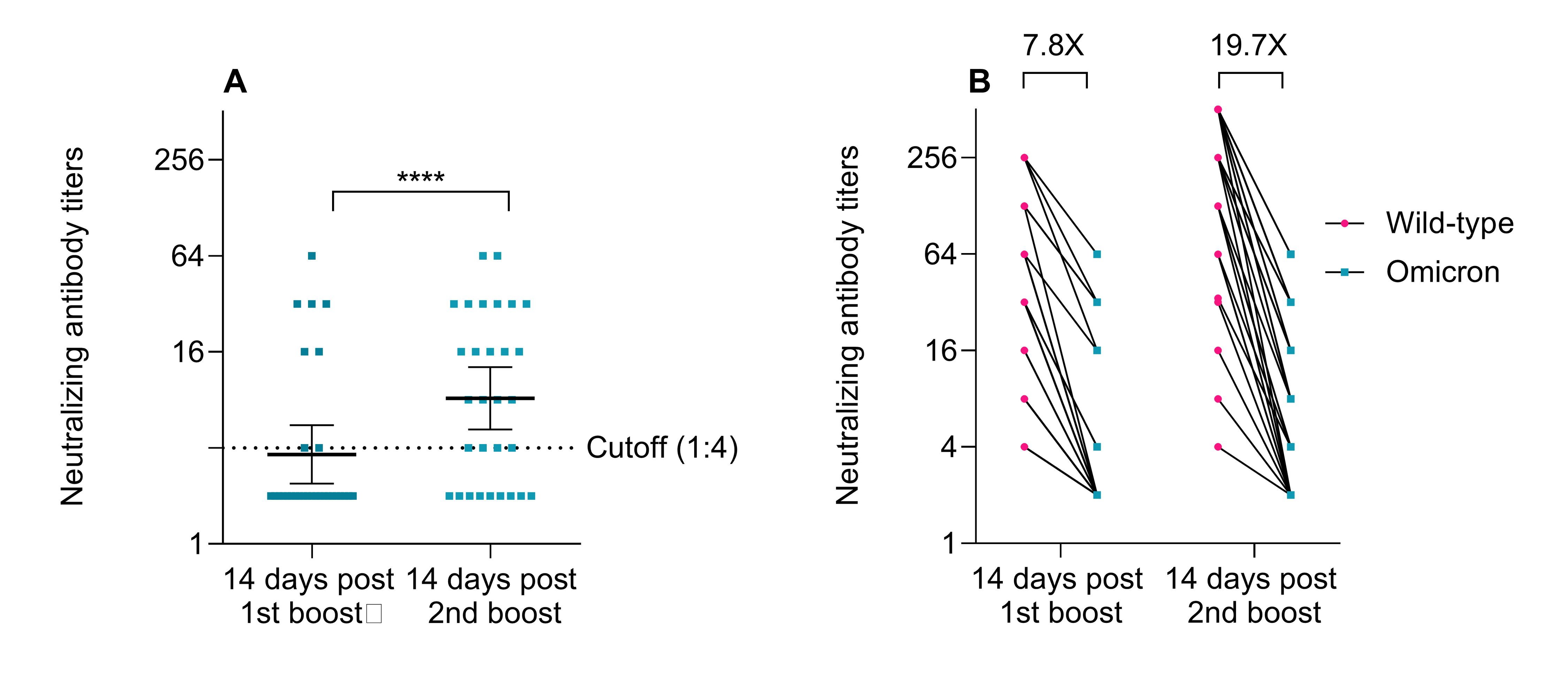

Supplement: S5 Fig — (A) GMTs of neutralizing antibodies against the Omicron variant 14 days post first and second boost. (B) Geometric mean ratios of neutralizing antibodies against wild-type relative to Omicron 14 days post first and second boost. The numbers indicate the geometric mean ratios. Cutoff (1:4) refers to the detection limit. Horizontal bars show geometric mean titer and error bars show 95% CI. CV/ZF/ZF (D0-D56-M6) = receiving Convidecia/ZF2001/ZF2001 at day 0, day 56, and month 6. ****P < 0.001. CI, confidence interval; GMT, geometric mean titer. (TIF) [file pmed.1003953.s010.tif]
