# Supplementary material for: Safety and immunogenicity of heterologous boost immunization with an adenovirus type-5-vectored and protein-subunit-based COVID-19 vaccine (Convidecia/ZF2001): A randomized, observer-blinded, placebo-controlled trial
Source: PLoS Med. 2022 May 26;19(5):e1003953. doi: 10.1371/journal.pmed.1003953 (PMC9187065; doi:10.1371/journal.pmed.1003953)
Supplement: Attachment — Submitted filename: 0-Response to reviewers-0408.docx [file pmed.1003953.s011.docx]

**Responses to the Comments from editors and reviewers.**

I think the authors need to include data on omicron responses. Delta is largely irrelevant now, as is the original wuhan variant.

**Response:** Thank you for your suggestions. We tested the serum samples for neutralizing antibodies against the omicron variant in CV/ZF/ZF (D0-D56-M6) regimen. Please see the results in line 437-446 of page 24 and S5 Fig.

“In addition, we tested the serum samples for neutralizing antibodies against the omicron variant in CV/ZF/ZF (D0-D56-M6) regimen. The results showed that 8 (27.6%) of 29 serum samples 14 days post 1st boost and 21 (70.0%) of 30 serum samples 14 days post 2nd boost were positive for neutralizing antibodies against the omicron variant (BA.1.1). The 2nd boost vaccination induced higher neutralizing antibodies against Omicron than that elicited by 1st boost, with the GMT of 8.2 (95%CI 5.2 to 12.8) vs 3.6 (2.4 to 5.5) (p＜0.001). The titer of neutralizing antibodies against the omicron variant was lower than that against the wild-type SARS-CoV-2 by a factor of 7.8 (95%CI 5.4 to 11.2) 14 days post 1st boost, and 19.7 (13.6 to 28.6) 14 days post 2nd boost (S5 Fig).”

The authors also need to provide a more consistent use of statistics and significance.
**Response:** We have revised the main text to keep the consistent of statistics and significance.

Requests from the editor:

The Data Availability Statement (DAS) requires revision: a study author cannot be the contact person for the data. Please direct data requests to a non-author institutional point of contact, such as a data access or ethics committee.
**Response:** We have revised the Data Availability Statement (DAS) requires. Direct data requests to the ethics committee ([ec@jscdc.cn](mailto:ec@jscdc.cn)). In addition, all relevant data are within the manuscript and its Supporting Information files.

Please report your abstract according to CONSORT for abstracts, following the PLOS Medicine abstract structure (Background, Methods and Findings, Conclusions) http://www.consort-statement.org/extensions?ContentWidgetId=562
**Response:** We have revised the abstract. Please see pages 3-5.

Please state that analysis was intention to treat in the Abstract.

**Response:** We have stated in the Abstract that the primary analysis was done in the intention-to-treat population. Please see line 48 of page 4.

At this stage, we ask that you include a short, non-technical Author Summary of your research to make findings accessible to a wide audience that includes both scientists and non-scientists. The Author Summary should immediately follow the Abstract in your revised manuscript. This text is subject to editorial change and should be distinct from the scientific abstract. Please see our author guidelines for more information: <https://journals.plos.org/plosmedicine/s/revising-your-manuscript#loc-author-summary>.

**Response:** We have added a short Author Summary. Please see Page 6.

Please place citations within square brackets throughout the manuscript.
**Response:** We have placed citations within square brackets.

Thanks for providing a CONSORT checklist. When completing the checklist, please use section and paragraph numbers, rather than page numbers.

**Response:** We revised the CONSORT checklist. Please see S1 CONSORT checklist.

Please cite your CONSORT checklist in the Methods section (‘This study is reported as per the Consolidated Standards of Reporting Trials (CONSORT) guideline (S1 Checklist)’ or similar). Similarly, please cite your Protocol/Analysis Plan in the relevant section of the Methods.

**Response:** We have cited the study protocol and the CONSORT checklist in the Methods section. Please see lines 151-152 of page 9.

In accordance with ICMJE requirements, PLOS Medicine requires prospective, public registration of a data sharing plan (as part of mandatory clinical trials registration) for all clinical trials that began enrollment on or after January 1, 2019.

**Response:** We have submitted IPD sharing statement to ClinicalTrials.gov (NCT04833101).

The description of the exclusion criteria (Lines 137-141) differ slightly from that presented in Supplementary Table 5 and the clinical trial registry (e.g. previous COVID-19 diagnosis). Please explain this discrepancy.
**Response:** We recruited participants from a population cohort receiving Convidecia.

In this cohort, subjects had no history of SARS-CoV-2 infection before priming with Convidecia, confirmed by SARS-CoV-2 IgM/IgG antibodies rapid test kit (Vazyme, Nanjing, China). Additionally, there was no SARS-CoV-2 epidemic in trial site during the study, so “previous COVID-19 diagnosis or SARS-CoV-2 infection” was not included in the exclusion criteria. We have added the statement in Procedures Section.

Please see lines 214-217 of page 12.

“We recruited participants from a population cohort receiving Convidecia, in which subjects had no history of SARS-CoV-2 infection before priming with Convidecia, confirmed by SARS-CoV-2 IgM/IgG antibodies rapid test kit (Vazyme, Nanjing, China).”

Please define "lost to follow-up" as used in this study. Other reasons for exclusion should be defined.
**Response:** We have defined the reasons to discontinue from the trial in the legend of Fig 1.

“Seven participants discontinued follow-up after vaccination. The reasons for dropout are withdrawn consents for participation.”

Please report the significant difference in injection site pain detailed in the legend of Table 2 in the main text.
**Response:** Thank you for your comments. We have reported the significant difference in injection site pain in the main text. Please see lines 320-322 of Page 18.

“The incidence of injection-site pain was higher in CV/ZF/ZF (D0-D28-M5) regimen than that in CV/ZF/ZF (D0-D56-M6) regimen (32.5% vs 7.5%, p=0.005). ”

The text in Figures 2-5 is too small to read, please enlarge.

**Response:** We have enlarged the size of the text to 12 in Figures 2-5.

Consider using a different colour scheme in Figure 2 panels A and B to avoid creating problems for those with red/green colour blindness.

**Response:** Thank you for your suggestions. We have adjusted the color scheme of figures.

Throughout, where p values are lower than 0.001, please report as P<0.001. Please also consistently report p values to 3 decimal places.
**Response:** We have modified as requested. Please see the Results section and Tables B-E in S1 Data.

Table S3: I could not see the corresponding asterisk for the footnote anywhere in the table.

**Response:** We have added the corresponding asterisk. Please see Table C in S1 Data.

Throughout the results, when stating a significant difference, please include the quantitative data and relevant p-value (e.g. line 296).
**Response:** We have now included the quantitative data and relevant p-value. Please see lines 320-322 of page 18 and line 441-443 of page 24.

“The incidence of injection-site pain was higher in CV/ZF/ZF (D0-D28-M5) regimen than that in CV/ZF/ZF (D0-D56-M6) regimen (32.5% vs 7.5%, p=0.005).”

“The 2nd boost vaccination induced higher neutralizing antibodies against Omicron than that elicited by 1st boost, with the GMT of 8.2 (95%CI 5.2 to 12.8) vs 3.6 (2.4 to 5.5) (p＜0.001).”

Table S1 is incorrectly labelled Table 1.
**Response:** Thank you very much for your careful checking. We have revised it.

Line 373: Please consider tempering the statement ‘is safe’ to ‘was not associated with safety concerns in this study’, or similar.
**Response:** Thank you for your suggestion. We have revised the statement ‘is safe’ to ‘is not associated with safety concerns in this study’. Please see lines 477-478 of Page 26.
“Our findings show that heterologous immunization of ZF2001 in individuals primed with Convidecia is not associated with safety concerns in this study.”

Line 437: ‘national vaccine programmes’.
**Response:** Thank you so much for your careful reading, we have revised it in the revised version. Please see line 556 of Page 29.

The Author Contributions, Conflict of Interest statement, and Data Availability statements can all be removed from the main text as these are captured as metadata via the Submissions Form.
**Response:** We have removed it from the main text.

Comments from the reviewers:

Reviewer #1:
"Safety and immunogenicity of heterologous boost immunization with an 2 adenovirus type-5-vectored and protein-subunit-based COVID-19 vaccine 3 (Convidecia/ZF2001): a randomized, observer-blinded, placebo-controlled trial"

In General
This manuscript describes an heterologous boosting study of an adenovirus-based vaccine (Convidecia) and an adjuvanted protein subunit COVID-19 vaccine (ZF2001). Both vaccins are not used in western world countries, but mainly applicated in China, Ecuador, Malaysia, Pakistan, and Uzbekistan. This makes the manuscript less interesting for western countries, however from a scientific viewpoint and from a world perspective, this manuscript has added value.

Introduction
83 This is correct, but AZ is also an adenovirus based platform and its efficacy was also relatively low. The question is whether it is due to only 1 dose or due to the platform? From my point of view, the adeno platforms induce a lower, but longer lasting immune response.

**Response:** We agree with you. We have revised the statement. Please see lines 109-112 of page 7.

“Compared with mRNA vaccines and protein-subunit-based vaccines containing novel adjuvants against COVID-19 (e.g. BNT162b2, mRNA-1273, NVXCoV2373), adenovirus-vectored vaccines (e.g.ChAdOx1 nCoV-19, Ad26.COV2-S and Ad5 nCoV) showed a relatively lower immunogenicity and efficacy against symptomatic disease”

85 With the waning  As the waning….

**Response:** Thank you very much for your careful checking. We have revised it. Please see line 113 of page 7.

88 Please let a native english speaker improve the grammar.

**Response:** Thank you for your suggestion. We have polished the English spelling and grammar of the manuscript.

94 Based on the both the concerns of  remove the first "the"
**Response:** Thank you very much for your careful checking. We have revised it. Please see line 122 of page 8.

Methods
* A figure showing the set up of the protocol would make it easier to read. Or make a new figure (preferred) or refer to figure 1.

**Response:** Thank you for your suggestion. We have added a figure showing the time point of injection and sample collection in Fig 1A.

123 This is the first time you use the abbreviation TIV.

**Response:** We have added the full name of the abbreviation TIV, with trivalent inactivated influenza vaccine (TIV).

121 I read this as 28 or 56 days after priming. Is this correct? Please clearify

**Response:** We have modified the sentences. Please see lines 153-156 of Page 9.

“In the original protocol, participants who have received one dose of Convidecia were randomly assigned (2:1) to receive either 1 intramuscular dose of ZF2001 or placebo control (trivalent inactivated influenza vaccine, TIV), administered at either 28 days or 56 days after priming.”

129 please consider to rephrase these sentences. If you use the words after priming you can state 1 and 5 months after priming.

**Response:** Thank you for your suggestions. We have modified the sentences. Please see lines 158-164 of pages 9-10.

“Four permutations of prime-boost schedule were investigated, including receiving ZF2001/ZF2001 at 28 days and 5 months after priming with Convidecia (referred to as CV/ZF/ZF (D0-D28-M5)), receiving ZF2001 at 5 months after priming (referred to as CV/ZF (D0-M5)), receiving ZF2001/ZF2001 at 56 days and 6 months after priming (referred to as CV/ZF/ZF (D0-D56-M6)), receiving ZF2001 at 6 months after priming (referred to as CV/ZF (D0- M6)).”

131 and further.. I would prefer to put the approval in the beginning of the methods section.

**Response:** Thank you for your suggestions. We have put the statement about the approval of study protocol in the beginning of the methods section. Please see lines 147-152 of page 9.

“The trial protocol was reviewed and approved by the institutional review board of the Jiangsu Provincial Center of Disease Control and Prevention (approval number: JSJK2021-A005-02) and performed in accordance with the Declaration of Helsinki and Good Clinical Practice. This trial was registered with ClinicalTrials.gov NCT04833101. The study protocol, including the CONSORT checklist, can be found in S1 Study Protocol and S1 CONSORT Checklist.”

138 Sars-cov infection  was this self-reported or also immunological confirmed?

**Response:** We recruited participants from a population cohort receiving Convidecia.

In this cohort, subjects had no history of SARS-CoV-2 infection before priming with Convidecia, confirmed by SARS-CoV-2 IgM/IgG antibodies rapid test kit (Vazyme, Nanjing, China). We have added the statement in Procedures Section.

Please see lines 214-217 of page 12.

“We recruited participants from a population cohort receiving Convidecia, in which subjects had no history of SARS-CoV-2 infection before priming with Convidecia, confirmed by SARS-CoV-2 IgM/IgG antibodies rapid test kit (Vazyme, Nanjing, China).”

150 The control influenza vaccine  did I miss something? Influenza vaccine? I have not read about this before? Or do you mean placebo?
157 This is the first time I understand that influenza vaccine is used as placebo. Please clarify this earlier. Please address in the introduction why an influenza vaccine was used and not a regular placebo.

**Response:** Yes, influenza vaccine was used as a placebo comparator rather than a saline placebo to maintain masking of participants and ensure that all participants obtain some benefit. We have added the reasons to select influenza vaccine as placebo control in Randomization and masking section. Please see lines 204-206 of page 12.

“Influenza vaccine was used as a placebo comparator rather than a saline placebo to maintain masking of participants and ensure that all participants obtain some benefit.”

173 grade is missing an e (2x)

**Response:** Thank you very much for your careful checking. We have revised it.

176 Please rephrase using less words and making it more simple to read. Please consider to put this is in the figure I was mentioning earlier.

**Response:** Thank you for your suggestions. We have added a figure showing immunization schedule and sample collection in Fig 1.

192 Live virus  which variant? I see you mention it in 194. Please try to write more compressed.

**Response:** Immunological primary and secondary outcomes were live virus neutralizing antibodies to wild-type SARS-CoV-2. We have revised it. Please line 265 of page 14.

201 Unfortunately I am not a virologist. However, I was told (the SARS-CoV-2 virus strain in Vero-E6 cells) that growing SARS COV on vero changes the virus and would not reflect real world data. Please comment on this and consider to put this consideration in the discussion.

**Response:** SARS-CoV-2 propagation on VeroE6 cells could lead to mutations or deletions in the multibasic cleavage site in the S protein. As we known, Vero-E6 cells were used in vitro neutralization assays to test the neutralizing antibodies to SARS-CoV-2 in most studies. Additionally, we used the WHO international standard as the reference with the serum samples measured in this study for calibration and harmonization of the serological assays.

198 Please consider to put the Immunogenicity assay-section in the supplemental file.

**Response:** Thank you for your suggestions. We have removed the immunogenicity assay-section to the supplemental materials. Please see S2 Text.

242 I am not a statistician. This section seems ok for me. But I hope someone else can check as well.

Results
273 Was the external comparator group also mentioned in the protocol submitted? If not, please replace to the discussion section.

**Response:** Thank you for your suggestions. The external comparator group was not predefined in the protocol. We have replaced to the discussion section. Please see line 558-562 of pages 29-30.

“Although we select two extend controls receiving homologous immunization of Convidecia following a 0-56 days regimen and 0-6 months regimen, which are both comparable with the cohorts receiving heterologous immunization between Convidecia and ZF001 in baseline characteristics (Table A in S1 Data), there may be some potential bias.”

284 the adverse events after priming were retrospectively determined? Is this stated clearly in the methods section?
288 38.5℃) after prime vaccination.  But I thought that priming was not part of the protocol?

**Response:** We recruited participants from a population cohort receiving Convidecia, in which safety data after priming with Convidecia were prospectively collected in the same methods as this study. We have stated it in the methods section. Please see lines 214-217 of page 12 and lines 227-228 of page 13.

### “We recruited participants from a population cohort receiving Convidecia, in which subjects had no history of SARS-CoV-2 infection before priming with Convidecia, confirmed by SARS-CoV-2 IgM/IgG antibodies rapid test kit (Vazyme, Nanjing, China).”

### “Safety data within 28 days after priming with Convidecia was collected in the same manner as the boost vaccination.”

294 Primary endpoint on immunogenicity is 14 days post boost  but you start with reporting 28 days post priming? Which is actually baseline?

### Response: The baseline was defined as day 28 after priming with Convidecia. Please see line 232-235 of page 13.

### “Blood samples were taken on day 28 (referred to as baseline), day 42, day 56, day 164 after priming in CV/ZF/ZF (D0-D28-M5) and CV/ZF/ZF (D0-M5) regimens, and on day 28 (baseline), day 56, day 70, day 84, day 194 after priming in CV/ZF/ZF (D0-D56-M6) and CV/ZF/ZF (D0-M6) regimens.”

298 In the vaccine group  not clear for me which group you mean. Control group?

**Response:** We have revised it. Please see lines 343-346 of pages 19-20.

“At 14 days post 1st boost, GMTs of neutralizing antibodies in recipients receiving ZF2001 28 days and 56 days apart was 18.7 (95% CI 13.7 to 25.5) and 25.9 (17.0 to 39.3), respectively, with geometric mean ratios of 2.4 (1.2 to 3.5) and 3.4 (1.8 to 6.4) compared to TIV.”

* Why was no blood drawn on the days of boosting? I cannot see this data in the figures.

1、 **Response:** We collected blood samples on the day of 1st boost, which was day 28 after priming for CV/ZF/ZF (D0-D28-M5) and CV/ZF (D0-M5) regimen, and day 56 after priming for CV/ZF/ZF (D0-D56-M6) and CV/ZF (D0-M6) regimen, respectively. Please see the Table C in S1 Data. We did not collect blood samples on the day of the 2nd boost with ZF2001. We have stated the limitation in the Discussion section. Please see lines 568-570 of page 30.

“we did not collect blood samples before the 3rd dose to assess the true impact of the 3rd dose, and it would explain why there was little difference between 2-dose and 3-dose regimens.”

* For the whole manuscript I see some sloppy typing errors. E.g in figure 5A Wide type instead of wilde.

**Response:** Thank you for your careful checking. We have polished the English spelling.

305-315 this section does not read very easily. Please try to simplify

### Response: We have revised this section. Please see lines 343-354 of pages 19-20.

“At 14 days post 1st boost, GMT of wild-type virus neutralizing antibodies in recipients receiving ZF2001 28 days and 56 days post priming was 18.7 (95% CI 13.7 to 25.5) and 25.9 (17.0 to 39.3), respectively, with geometric mean ratios of 2.4 (95% CI 1.2 to 3.5) and 3.4 (1.8 to 6.4) compared to TIV. ZF2001 given as the 2nd boost dose induced comparable neutralizing antibodies to wild-type SARS-CoV-2 in four prime-boost regimens, with GMTs at 14 days post boost of 107.2 (73.7 to 155.8) in CV/ZF/ZF (D0-D28-M5) regimen, 90.5 (45.6 to 179.8) in CV/ZF (D0-M5) regimen, 141.2 (83.4 to 238.8) in CV/ZF/ZF (D0-D56-M6) regimen and 94.1 (44.0 to 200.9) in CV/ZF (D0-M6) regimen, respectively (Fig 2A, Fig 2B, Table C in S1 Data). The GMFIs ranged from 8.9 (95%CI 4.8 to 16.7) in CV/ZF (D0-M6) regimen to 17.3 (11.1 to 26.9) CV/ZF/ZF (D0-D56-M6) regimen when compared to baseline (28 days post prime) (Fig 2C, Table C in S1 Data).**”**

317 was this comparison with homologous boosting predefined in the protocol?

**Response:** This was an ad hoc analysis which was not predefined in the protocol. We have state it in the Procedure section. Please see lines 251-255 of page 14.

“An ad hoc analysis was conducted, comparing the wild-type virus neutralizing antibodies to a sample serum panel from 40 participants receiving homologous immunization of Convidecia following a 0-56 days regimen and 20 participants receiving homologous prime-boost vaccination of Convidecia with a 0-6 months regimen in previous trials”

322 predefined comparison on age?

**Response:** Yes, randomization was stratified by age (18-59 years and ≥60 years) at time of enrollment, so the subgroup analysis was performed according to age. Please see Fig 3 and Table D in S1 Data.

328 CV/ZF/ZF  CF? see other groups

**Response:** Thank you for your careful checking. We have revised it.

333 For me it is confusing that you are now looking at 14 days after second boost!!!!
Try to make it easy for the reader to understand. See also 347…. I thought primary endpoint was 14 days post first boost?

**Response:** In this study, the primary endpoint was wild-type virus neutralizing antibodies 14 days post each boost. The results of wild-type virus neutralizing antibodies showed that heterologous vaccination with Convidecia and ZF2001 at an interval of 5 months or 6 months induced higher antibody titers than that elicited by immunization 28 days or 56 days apart. We thought antibody responses were more interesting 14 day after 2nd boost than 1st boost, and presented anti-RBD and anti-S IgG 14 days post 2nd boost in main text. We have added the results of binding antibodies 14 days post 1st boost. Please see lines 395-399 of page 22.

“In line with wild-type virus neutralizing antibodies, ZF2001 induced significantly higher anti-RBD IgG and anti-spike IgG 14 days post 1st boost, compared with TIV (Fig 4). Anti-RBD IgG GMRs between ZF2001 and TIV were 5.2 (2.6, 10.4) in “D0-D28” regimen and 9.3 (4.3, 20.4) in “D0-D56” regimen. GMRs for anti-spike IgG were consistent with those of anti-RBD IgG (Table E in S1 Data).”

345 remove delta  this section is also about wilde type.
**Response:** This subsection is about neutralizing antibodies to the delta variant. Please see line 427 of page 23.

Discussion
375 Do you think that 56 days post priming gives less AE than 28 days post priming?

Response: The safety results showed that the frequency of systemic adverse reactions within 28 days after boost immunization with ZF2001 was lower than that after priming with Convidecia.

382 "we founded"  we found

**Response:** Thank you for your careful checking. We have revised it.

416 "not increases the cellular immunity responses "  Tom y opinion this has to do with the lab method (Elispot). In our analysis we also see a plateau in the elispot, but a clear further grow when looked at t-cells with a different method.
**Response:** Thank you for your comments. We have added the potential effect of Elispot on the results of cellular immunity responses in Discussion section. Please see lines 540-543 of page 29.

“The lack of further increase in T cell responses in our study might be related to the test method, since T cells detected would not continue to increase after reaching a plateau in the ELISpot assay.”

@ What happened with participants that became SARS-cov positive during the trial?
I have not read anything I think
**Response:** There was no SARS-CoV-2 epidemic in trial site during the study. If a subject develops fever accompanied by respiratory symptoms, he/she will be instructed to seek medical attention and notify study staff. For the suspected COVID-19, the participant will have a nasal/throat swab taken for PCR test. We added the methods for ascertaining the COVID-19 in Procedures Section. Please see lines 228-231 of Page 13**.**

Reviewer #2: Statistical review
This paper reports a randomised trial assessing safety and immunogenicity of heterologous boost vaccination.
Generally the trial is reported well. I have some minor comments.
1. Abstract: Without looking at the main paper, I found it quite hard to follow the different groups in the trial from the abstract. I would recommend emphasising early that there were four groups to help the reader follow the subsequent text.
**Response:** Thank you for your suggestions. We have revised the Abstract. Please see pages 3-5.

2. Abstract: as the primary outcome, it might be good to comment on whether there were differences in the adverse reactions between group (or even better, provide the number per group).
**Response:** Thank you for your comments. We have added the results of differences across the groups. Please see lines 52-57 of page 4.

“A total of 26 participants (21.7%) reported solicited adverse events within 7 days post boost vaccinations, and all the reported adverse reactions were mild, with 13 (32.5%) in CV/ZF/ZF (D0-D28-M5) regimen, 7 (35.0%) in CV/ZF (D0- M5) regimen, 4 (10.0%) in CV/ZF/ZF (D0-D56-M6) regimen, and 2 (10.0%) in CV/ZF (D0-M6) regimen, respectively.”

3. Abstract: as the trial was powered to detect superiority in GMT of neutralizing antibodies, I would recommend this analysis is summarised in the abstract (preferably with estimated difference, CI and p-value).
**Response:** Thank you for your suggestions. We have revised the Abstract. Please see line 57-60 of page 4.

“At 14 days post 1st boost, GMTs of neutralizing antibodies in recipients receiving ZF2001 28 days and 56 days apart was 18.7 (95% CI 13.7 to 25.5) and 25.9 (17.0 to 39.3), respectively, with geometric mean ratios of 2.0 (1.2 to 3.5) and 3.4 (1.8 to 6.4) compared to TIV.”

4. Randomisation and masking: was stratified block randomisation used?
**Response:** Yes, stratified block randomisation was used for the randomisation, and the size of block is six. Please see lines 201-204 of page 11.

“Participants were stratified by age (18-59 years and ≥60 years), and then randomly assigned (2:1) to vaccine group receiving ZF2001 and “placebo” group receiving a trivalent inactivated influenza vaccine (TIV) either 28 days or 56 days apart. Randomization was done by random block size of six.”

5. Outcomes: the clinicaltrials.gov page lists some outcomes involving the proportion of patients with at least four-fold increase, which I do not see mentioned in this paper.
**Response:** Seroconversion was defined as at least a fourfold increase in the antibody titers at different time points after boost immunization compared to baseline level (at 28 days post prime dose) in the Procedure section. The results of seroconversion rate were presented in line 372-375 of page 21 in main text and Fig 2D.

“At 14 days post the 2nd boost, the seroconversion of neutralizing antibody titers were observed in 90.0% (36/40) of the participants in CV/ZF/ZF (D0-D28-M5), and 89.5% (17/19) in CV/ZF (D0-M5), and 91.7% (33/36) in CV/ZF/ZF (D0-D56-M6) and 83.3% (15/18) in CV/ZF (D0-M6) regimen, respectively (Fig 2D, Table C in S1 Data).”

6. Results, line 304: it's not clear what these two p-values represent. It would be good to have the estimated difference between arms and CI, either in the text or in a table. Figures 2-4 are good but do not give the differences between arms.
**Response:** We have revised the sentence and added labels showing the differences between groups in figures. Please see line 343-346 of pages 19-20 and revised Figures 2-4.

“14 days post 1st boost, GMTs of wild-type virus neutralizing antibodies in recipients receiving ZF2001 28 days and 56 days apart was 18.7 (95% CI 13.7 to 25.5) and 25.9 (17.0 to 39.3), respectively, with geometric mean ratios of 2.0 (1.2 to 3.5) and 3.4 (1.8 to 6.4) compared to TIV.”
Reviewer #3:

The manuscript by Jin et al., describes a heterologous boosting strategy for individuals previously vaccinated with one dose of the Adenovirus-vectored vaccine Convidecia, who then received with 1 to 2 doses of the protein subunit vaccine FZ2001. They report the reactogenicity and immunogenicity of the booster doses against ancestral virus and the Delta variant. They show the heterologous primer-boost strategy is safe and well tolerated, and generates a humoral and cellular response.

There are some points that need to be addressed to ensure the manuscript is at the level required for this journal:
Currently there are already a number of commercially available vaccines based on the ancestral Wuhan variant, and numerous reports of heterologous mixing and matching of doses. A current issue in the field is a lack of standardisation across assays to measure neutralising antibody titres. Jin et al., should be commended by addressing this via incorporation of the WHO reference standards, which allow them to report titres in IU/ml. However, despite this there was no real comparison provided to other commercially vaccines which makes it difficult to determine the level of protection this prime-boost strategy would afford, and therefore if it would offer any real benefit compared to other already approved vaccines. In addition, they show that this heterologous prime-boost strategy performs no better (in terms of neutralising antibody titres) than a homologous prime boost with Convidecia at 6 months.
Further analysis to estimate the efficacy of this prime-boost schedule (for example based on the model provided by Khoury et al., Nature Med) would significantly strengthen the manuscript.
Other studies using mRNA-based vaccines have shown that a 3rd dose at 6-months leads to large increases in neutralising antibody titres compared to pre-boost titres. As pre-3rd dose neutralisation titres were not determined, it is difficult to assess the true impact of the 3rd dose.  I.e. Had titres for all individuals had fallen to below detectable levels, and could this explain why there was little difference between the 2-dose versus 3-dose regimen. This information would also provide data on the decay rate of antibodies titres over time.

Inclusion of neutralising antibodies against Omicron would also add to the value of the paper.

**Response:** Thank you for your comments.

1、In this study, the WHO international standard for anti-SARS-CoV-2 IgG (NIBSC code 20/136) was used side by side as reference for calibration and harmonization of the serological assays. Please see Table C in S1 Data. Our results showed that four prime-boost regimens with Convidecia and ZF2001 induced robust immune responses, with neutralizing antibody GMTs ranged 283 to 441 IU/ml. This is comparable to 344 IU/ml in participants primed with Janssen Ad26.COV2-S and then given BNT162b2 (Atmar RL, et al. N Engl J Med, 2022). We have stated it in the Discussion section. Please see lines 480-485 of page 26.

“Four prime-boost regimens induced robust immune responses, with GMTs of wild-type neutralizing antibodies ranged 91 to 141, which is equivalent to range 283 to 441 IU/ml using the World Health Organization (WHO) international standard. This is comparable to 344 IU/ml in participants primed with Janssen Ad26.COV2-S and then given BNT162b2”

2、 Khoury et al. and Feng S et al. provided models to identify correlates of protection against SARS-CoV-2, which was very interesting for the estimate of vaccine efficacy. However, we would not directly extrapolate the efficacy to immunogenicity data of prime-boost schedules due the lack of model parameters.
3、 We did not collect the blood samples before the 3rd dose with ZF2001. We acknowledge that this is a limitation and have stated it in the Discussion section. Please see lines 568-570 of page 30.

“we did not collect blood samples before the 3rd dose to assess the true impact of the 3rd dose, and it would explain why there was little difference between 2-dose and 3-dose regimens.”

4、 We have added to measure the neutralising antibodies to the Omicron variant in recipients receiving CV/ZF/ZF (D0-D56-M6) regimen. Please see lines 437-446 of page 24 and S5 Fig.

“In addition, we tested the serum samples for neutralizing antibodies against the omicron variant in CV/ZF/ZF (D0-D56-M6) regimen. The results showed that 8 (27.6%) of 29 serum samples 14 days post 1st boost and 21 (70.0%) of 30 serum samples 14 days post 2nd boost were positive for neutralizing antibodies against the omicron variant (BA.1.1). The 2nd boost vaccination induced higher neutralizing antibodies against Omicron than that elicited by 1st boost, with the GMT of 8.2 (95%CI 5.2 to 12.8) vs 3.6 (2.4 to 5.5) (p＜0.001). The titer of neutralizing antibodies against the omicron variant was lower than that against the wild-type SARS-CoV-2 by a factor of 7.8 (95%CI 5.4 to 11.2) 14 days post 1st boost, and 19.7 (13.6 to 28.6) 14 days post 2nd boost (S5 Fig).”

Minor points:

Line 39, should read Convidecia/ZF2001/ZF2001?

**Response:** Thank you for your careful checking. It should be Convidecia/ZF2001/ZF2001.

Individuals with a previous diagnosis of SARS-CoV-2 were excluded, however were initial serum samples screened for anti- N or M antibodies to confirm none of the individuals had an asymptomatic infection?

**Response:** There was no SARS-CoV-2 epidemic in trial site during study. We recruited participants from a population cohort receiving Convidecia. In this cohort, subjects had no history of SARS-CoV-2 infection before priming with Convidecia, confirmed by SARS-CoV-2 IgM/IgG antibodies rapid test kit (Vazyme, Nanjing, China). We have added the statement in Procedures Section.

Please see lines 214-217 of page 12.

“We recruited participants from a population cohort receiving Convidecia, in which subjects had no history of SARS-CoV-2 infection before priming with Convidecia, confirmed by SARS-CoV-2 IgM/IgG antibodies rapid test kit (Vazyme, Nanjing, China).”

In table 2, it is not clear if the adverse reactions are post first or second boost.

**Response:** In table 2, the data presented are the adverse reactions post first and second boost. We have revised Table 2.

Line 298, "in the vaccine group" should be clarified to state which vaccine.

**Response:** We have revised the sentence. Please see lines 343-346 of pages 19-20.

“14 days post 1st boost, GMTs of wild-type virus neutralizing antibodies in recipients receiving ZF2001 28 days and 56 days apart was 18.7 (95% CI 13.7 to 25.5) and 25.9 (17.0 to 39.3), respectively, with geometric mean ratios of 2.0 (1.2 to 3.5) and 3.4 (1.8 to 6.4) compared to TIV.”

Further information on the ZF2001 would assist the reader, i.e. how is it produced
**Response:** We have added information on ZF2001 in Vaccines subsection. Please see lines 192-195 of page 11.
“ZF2001 encodes the SARSCoV-2 RBD antigen (residues 319–537, accession number YP_009724390), with two copies in tandem repeat dimeric form, and was manufactured in the CHOZN CHO K1 cell line as a liquid formulation containing 25 μg per 0·5 mL in a vial, with aluminium hydroxide as the adjuvant.”

Reviewer #4: Huge amount of different studies are going on around the world to research COVID-19 vaccines. Today it is obvious that it is necessary to use booster doses of vaccines, especially in the face of the emergence of new variants of the virus.
The authors conducted the study to investigate the safety and immunogenicity of heterologous immunization prime with Ad5-based vector vaccine and boost with RBD-subunit vaccine. The authors provide detailed description of the current state of COVID-19 vaccines development and administration. The provided Protocol of the trial was changed because investigators decided to add additional boost vaccination with protein-subunit vaccine at 4 months after the first boost dose.
As the study was analyzed, a number of questions and comments arose, which are summarized below:
1. The authors investigated several administration regimens of vaccines. One of these schemes included the injection with Convidecia at day 0, the first boost immunization with ZF2001 and the second boost immunization with ZF2001 at 4 months after. This regimen is referred throughout the manuscript as CV/ZF/ZF (D0-D28-M5). However in the abstract it is referred as Convidecia/Convidecia/ZF2001 (Line 39). It should be corrected.

**Response:** Thank you very much for your careful checking. We have revised the Abstract. Please see pages 3-5.

2. Methods and Findings in Abstract - it is not very clear from the description how many groups of vaccinated people there were and in what regimens the vaccines were administered. The authors abbreviate the names of vaccines, while in one place to two letters (lines 40-41), in another to one (lines 55-56). Abbreviations should be brought to uniformity.

**Response:** We have revised the Abstract. Please see page 3-5.

3. Lines 113-114 - Authors should add a description of which antigens are included in the subunit vaccine.

**Response:** We have added the description of antigens included in the subunit vaccine. Please lines 138-141 of pages 8-9.

“Here, we present safety and immunogenicity of a heterologous prime-boost vaccination of a recombinant adenovirus type-5-vectored COVID-19 vaccine (Convidecia) followed by a recombinant protein-subunit-based COVID-19 vaccine composed of dimetic receptor-binding domain (RBD) (ZF2001) in healthy adults.”

4. Line 123 - it is necessary to decipher the abbreviation TIV (decoding is given only on line 157).

**Response:** We have added the full name of the abbreviation TIV, with trivalent inactivated influenza vaccine (TIV).

5. Line 173 - «grad 1» and «grad 2» - need correction to «grade 1» and «grade 2».

**Response:** Thank you very much for your careful checking. We have revised it.

6. Lines 188-193 - The authors describe the immunological studies. The question arises whether the authors analyzed the presence of antibodies specific to the N protein of the SARS-CoV-2 virus in volunteer's sera before the administration of the subunit vaccine and the influenza vaccine. There is no information about this analysis in the protocol. If this analysis was not performed, this should be stated in the study limitations.

**Response:** In this trial, participants were recruited from a population cohort receiving Convidecia, in which subjects had no history of SARS-CoV-2 infection before priming with Convidecia, confirmed by SARS-CoV-2 IgM/IgG antibodies rapid test kit (Vazyme, Nanjing, China). Additionally, there was no SARS-CoV-2 epidemic in trial site during the study, so we did not test the antibodies specific to the N protein of the SARS-CoV-2 virus before the administration of the subunit vaccine and the influenza vaccine. We have added the statement in Procedures Section. Please see lines 214-217 of page 12.

“We recruited participants from a population cohort receiving Convidecia, in which subjects had no history of SARS-CoV-2 infection before priming with Convidecia, confirmed by SARS-CoV-2 IgM/IgG antibodies rapid test kit (Vazyme, Nanjing, China).”

7. Line 194 - The authors indicate that the delta variant was used for exploratory outcomes of neutralizing antibodies. Why weren't the results of the analysis of neutralizing antibodies against the wild-type virus (whose glycoprotein homologous to the vaccine) not used for this purpose?

**Response:** In this study, neutralizing antibodies against the wild-type virus was the primary outcomes of the immunogenicity.

8. Lines 252-256 - The authors give the criteria by which the statistical analysis was carried out. A number of questions arise. What method was used to evaluate the normality of the data distribution in the sample pools? What method was used to evaluate unpaired samples in the case of non-normal distribution? This information should be added to the description of statistical methods.

**Response:** Thank you for your suggestions. We have added the methods to test normal distribution of the data and analysis non-normal distributed data. Please see lines 289-291 of page 16.

“Normal distribution of the data was tested using Kolmogorov-Smirnov test. For non-normal distributed data, Wilcoxon rank-sum test was used.”

9. Lines 206-207 and Figure 2 - The authors describe the procedure for the microneutralization reaction, describing that the sera were titrated in two-fold steps from 1/8 to 1/256 (wild-type variant) and from ¼ to 1/128 (Delta variant). The figure also shows the level of neutralizing antibodies in units (not titers), and there is no information about what level of units corresponds to the presence of virus neutralizing antibodies. At the same time, there is no information in the text of the article on how the titer of virus-neutralizing antibodies was converted into units. This information should be added to the immunogenicity analysis section of the methods. There is also confusion when comparing data: the results of the analysis of virus-neutralizing antibodies against the wyld-type variant are given in units, while neutralizing antibodies against the Delta variant are given in titers. In order to limit misunderstanding, it is necessary to bring the results of the analysis to uniformity: either to titers or to units.

**Response:** We have added the level of the WHO International Standard corresponds to the presence of virus neutralizing antibodies in main text and revised Fig 2. Please see lines 243-244 of page 13 in Procedures subsection and Fig 2**.**

“The WHO reference serum 1000 IU/mL equivalent to live virus neutralizing antibody titer of 1:320 against the wild-type.”

To uniformity, we reported the result of live virus neutralizing antibodies in titers in main text and revised Figures.

10. Figure 5 - it is necessary to make an explanation in the description what the numbers above the brackets mean (0.35, 0.37, etc.)

**Response:** We revised the figure 5 **.**The numbers above the brackets indicate the geometric mean ratios of neutralizing antibody titers against wild-type relative to Delta. We have added the comment to the legend for Figure 5A and 5B.

11. Line 304 - The authors give p-values, but do not explain which data groups they refer to and by what criterion they were analyzed. Authors should add this information.

**Response:** We have revised the description of the sentence. Please see lines 343-346 of pages 19-20 in **Immunogenicity subsection.**

“14 days post 1st boost, GMTs of wild-type virus neutralizing antibodies in recipients receiving ZF2001 28 days and 56 days apart was 18.7 (95% CI 13.7 to 25.5) and 25.9 (17.0 to 39.3), respectively, with geometric mean ratios of 2.0 (1.2 to 3.5) and 3.4 (1.8 to 6.4) compared to TIV.”

12. Figure 2 AB, Figure 3, lines 293-323 - in the description of the results it is indicated that not all volunteers had virus-neutralizing antibodies detected. At the same time, when analyzing the figures, a different impression emerges. There is no information in the figures about the level at which units show the presence of virus-neutralizing antibodies, which can confuse the reader.

**Response:** We added the lower limit of detection in Figs2A and 2B, and Figure 3.

13. Lines 321-323 - Was the difference in amount of live virus neutralizing antibodies between different age groups statistically significant?

**Response:** Neutralizing antibodies 14 days after boost were numerically higher in participants aged 18-59 years than in those over 60 years across four regimens, but no significant difference were found between age subgroups due to small sample size. Please see lines 380-383 of page 21.

“Neutralizing antibodies 14 days after boost dose were numerically higher in participants aged 18-59 years than in those over 60 years across four regimens, but no significant difference were found between age subgroups due to small sample size (Fig 3, Table D in S1 Data).”

14. Line 357 - Variant B.1.617 indicated. It should be corrected to B.1.617.2.

**Response:** Thank you very much for your careful checking. We have revised it.

15. Lines 367-370 - The results of the analysis of the cellular response given in the text do not correspond to the results presented in Figure 5D. The results presented in this place completely copy the results indicated in lines 364-367. Data should be corrected

**Response:** Thank you very much for your careful checking. We have revised it. Please see lines 471-475 of pages 25-26.

“The median of IFN-γ spot counts per 106 PBMCs 14 days after 2nd boost was 30.0 (IQR: 0.0, 65.0) in CV/ZF/ZF (D0-D28-M5) regimen, 20.0 (IQR: 0.0, 60.0) in CV/ZF (D0-M5) regimen, 20.0 (IQR: 10.0, 55.0) in CV/ZF/ZF (D0-D56-M6) regimen and 10 (IQR: 0.0, 20.0) in CV/ZF (D0-M6) regimen, respectively.”

16. Lines 438-455 - In the limitations of the study, the authors should also indicate the relatively small sample size in some groups (groups of 10 volunteers - Table 1).

**Response:** Thank you for your suggestions. We have added the limitation of small sample size. Please see lines 570-572 of page 30.

“Lastly, the sample size of each regimen was relatively small, which limited the statistical power for age subgroup analyses. It is needed to be validated in a large sample size.”

General comments:
1. The authors need to check the correctness of the presentation of the results of the study so that the results described in the text correspond to the results presented in the figures.

**Response:** Thank you for your comments. We checked the full text to make sure the results to be consistent with that presented in the figures.
2. Authors need to bring the results of the study of neutralizing antibodies to uniformity. It is also necessary to indicate the detection limit of neutralizing antibodies in the figures so that the reader does not have a false opinion about the presence of antibodies in the blood serum of all volunteers.

**Response:** Thank you for your suggestions. We reported the result of live virus neutralizing antibodies in titers in main text and revised Figures and added the detection limit of neutralizing antibodies in the figures.

3. In the figures (or as an additional table in the appendix), it would also be useful to indicate the percentage of volunteers with a detectable level of neutralizing antibodies to both variants of the SARS-CoV-2 virus that were used in the study.
**Response:** Thank you for your suggestions. We have added the seropositive rate of neutralizing antibodies to wild-type virus and Delta variant in Table C in S1 Data.

In general, the authors present interesting data on the combination of vaccines based on different technological platforms: a vector vaccine and a subunit vaccine. The article can be accepted for publication after making corrections and eliminating inconsistencies.
