# Supplementary material for: Safety and immunogenicity of heterologous boost immunization with an adenovirus type-5-vectored and protein-subunit-based COVID-19 vaccine (Convidecia/ZF2001): A randomized, observer-blinded, placebo-controlled trial
Source: PLoS Med. 2022 May 26;19(5):e1003953. doi: 10.1371/journal.pmed.1003953 (PMC9187065; doi:10.1371/journal.pmed.1003953)
Supplement: Attachment — Submitted filename: 0-Response to reviewers-0509.docx [file pmed.1003953.s012.docx]

**Response to the comments from reviewers:**
Reviewer #2: Thank you to the authors for addressing my previous comments well. I have no further issues to raise.

Response: Thank you for your comments on the original manuscript.
Reviewer #3: The authors have addresses my previous concerns, however, the manuscript still needs editing to improve the grammar/correct typos.

Response: Thank you for your original comments on the manuscript. We have carefully examined the entire manuscript, and modified the grammar and spelling errors.
For example:
Line 121, thrombocytopenia is other challenge, should read "thrombocytopenia is another challenge"

Response: Thank you for your careful checking. We have revised it. Please see line 124 of the revised manuscript with changes marked.

“......but severe thrombotic events with thrombocytopenia is another challenge for adenovirus-based vaccines”
Line 208 Personals; should be personnel

Response: Thank you for your careful checking. We have revised it. Please see line 213 of the revised manuscript with changes marked.

“Personnel who prepared and administered vaccination were aware of group allocation,......”
The tense needs to be changed in lines 228-231 i.e "if a subject develops" should be if a subject developed etc.

Response: Thank you for your suggestion. We have revised it. Please see lines 232-235 of the revised manuscript with changes marked:
If a subject developed fever accompanied by respiratory symptoms, he/she was instructed to seek medical attention and notify study staff. For the suspected COVID-19, the participant had a nasal/throat swab taken for PCR test.
When referring to when the blood samples were taken (lines 232 onwards) it would be helpful to refer back to figure 1 in the text.

Response: Thank you for your suggestion. We have referred to Fig 1A in the text. Please see line 239 of the revised manuscript with changes marked.

“......and on day 28 (referred to as baseline), day 56, day 70, day 84, day 194 after priming in CV/ZF/ZF (D0-D56-M6) and CV/ ZF (D0-M6) regimens (Fig 1A)”
Line 265 should say wild-type SARS-CoV-2

Response: Thank you for your careful checking. We have revised it. Please see line 269 of the revised manuscript with changes marked.

“......and neutralization antibody titers against wild-type SARS-CoV-2”
Line 506 "As we known" should read as we know

Response: Thank you for your careful checking. We have revised it. Please see line 510 of the revised manuscript with changes marked.

“As we know,......”
Line 531 ZF2001 as a boost dose not; should read does not

Response: Thank you for your careful checking. We have revised it. Please see line 535 of the revised manuscript with changes marked.

“The use of ZF2001 as a boost does not further increase the cellular immunity responses.....”
In supplementary text 2 accession numbers/details for the omicron variant are missing.

Response: We have added accession numbers/details for the omicron variant:

“omicrom variant BA.1.1: hCoV-19/Jiangsu/JS01/2022(EPI_ISL_12511653).”
Reviewer #4: The authors made adjustments according to the comments received. When reviewing the article, one comment remained:
In the background, it is necessary to make a transcript in lines 32 and 33 - Convidencia (CV) and ZF2001 (ZF).

Response: We have revised it. Please see lines 32-33 of the revised manuscript with changes marked:
”We assessed the safety and immunogenicity of heterologous immunization with a recombinant adenovirus type-5-vectored COVID-19 vaccine (Convidecia, hereafter referred to as CV) and a protein-subunit-based COVID-19 vaccine (ZF2001, hereafter referred to as ZF).”
